# Efficacy and safety of SMET12 in combination with toripalimab and chemotherapy in advanced non-small-cell lung cancer patients tested positive for EGFR protein who are treatment-naïve or harbor acquired resistance to standard therapy: a phase 2, multi-cohort clinical trial

**DOI:** 10.3389/fimmu.2025.1706961

**Published:** 2026-01-08

**Authors:** Jinghui Lin, Shanshan Chen, Meifang Li, Lihong Weng, Haipeng Xu, Qiang Wang, Jing Zhang, Dong Lin, Haipo Wang, Qinying Liu, Zhiyong He

**Affiliations:** 1 Department of Medical Oncology, Clinical Oncology School of Fujian Medical University, Fujian Cancer Hospital, National Health Commission (NHC) Key Laboratory of Cancer Metabolism, Fuzhou, Fujian, China; 2 Fujian Provincial Key Laboratory of Tumor Biotherapy, Clinical Oncology School of Fujian Medical University, Fujian Cancer Hospital, National Health Commission (NHC) Key Laboratory of Cancer Metabolism, Fuzhou, Fujian, China

**Keywords:** Keywords: bispecific T cell engager, combination chemotherapy, efficacy, immunotherapy, non-small-cell lung cancer, safety, SMET12, toripalimab

## Abstract

**Objective:**

SMET12 is a bispecific T-cell engager targeting epidermal growth factor receptor (EGFR) and CD3. This phase 2 clinical trial aimed to investigate the efficacy and safety of SMET12 plus toripalimab and chemotherapy among advanced non-small-cell lung cancer (NSCLC) patients tested positive for EGFR protein, including treatment-naïve patients, patients with resistance to first-line immune checkpoint inhibitors-containing therapy and EGFR-mutated patients with resistance to first-line EGFR tyrosine kinase inhibitors (TKIs), and to examine the associations of lymphocyte numbers and differentiation patterns with therapeutic efficacy among advanced NSCLC patients.

**Methods:**

All advanced NSCLC patients were histologically diagnosed and tested positive for EGFR protein. This trial included three cohorts: Cohort A consisted of treatment-naïve patients, and Cohort B consisted of patients acquiring resistance to first-line immune checkpoint inhibitors-containing therapy, while Cohort C consisted of *EGFR*-mutated patients acquiring resistance to TKIs. All participants received triple therapy with SMET12, toripalimab and chemotherapy, and participants with complete response (CR), partial response (PR) or stable disease (SD) following 2 to 4 cycles of chemotherapy were given maintenance therapy with SMET12 plus toripalimab until progressive disease (PD) or unacceptable toxicity. Primary endpoints included safety and efficacy measures, including objective response rate (ORR), disease control rate (DCR), and progression-free survival (PFS). This trial was registered at https://clinicaltrials.gov (Trial registration number: NCT06208033).

**Results:**

A total of 32 patients were included in the trial until January 21, 2025. The ORR, DCR and median PFS were 83.3%, 100% and 8.3 (95% *CI*: 3.8, 12.8) months in Cohort A, 22.2%, 55.6% and 4.2 (95% *CI*: 3.6, 4.8) months in Cohort B, and 41.7%, 100% and 7.2 (95% *CI*: 5.0, 9.4) months in Cohort C. The most common treatment-related adverse events (20% incidence and higher) included cytokine release syndrome (53.13%), leukopenia (43.75%), elevated alanine aminotransferase (43.75%), neutropenia (37.5%), constipation (31.25%), and elevated aspartate aminotransferase (28.13%). Grade 3–4 adverse events included neutropenia (15.63%), Lung infections (15.63%), anemia (6.25%), immune-related hepatitis (6.25%), immune-related pneumonia (6.25%), leukopenia (3.13%), and hypertriglyceridemia (3.13%). No grade 5 adverse events were observed. At baseline among the three cohorts, Cohort A presented the lowest Treg, PD-1^+^ and LAG-3^+^T cell proportions; Cohort B had the highest Treg and LAG-3^+^T cell proportions, and Cohort C had the highest PD-1^+^T cell proportion. High numbers of peripheral blood T lymphocytes and CD69^+^ T cells, and higher proportions of central memory T cells (Tcm) and naïve T cells (Tn) may be predictive of a higher response to the triple therapy, and an increase in the positive rate of PD-1 and LAG3 expression on T cell surface and proportions of regulatory T cells (Tregs), effector memory T cells (Tem) and terminally differentiated effector T cells (Tte) may correlate with poor prognosis.

**Conclusions:**

Triple therapy consisting of SMET12, toripalimab, and chemotherapy exhibited manageable safety in patients with advanced non-small cell lung cancer (NSCLC) expressing EGFR protein, while demonstrating promising efficacy in EGFR-mutant patients with acquired resistance to EGFR-TKIs. The functional phenotype and differentiation pattern of peripheral blood T lymphocytes exert a critical impact on the therapeutic response to this triple therapy.

**Clinical trial registration:**

https://www.clinicaltrials.gov/study/, identifier NCT06208033.

## Introduction

Lung cancer is a leading cause of cancer-associated mortality across the world (1). Non-small-cell lung cancer (NSCLC) accounts for approximately 85% of all lung cancer cases, and the 5-year survival rate is only 8% among advanced NSCLC patients (2). Despite great strides of targeted therapy and immunotherapy in treatment of NSCLC (3–5), there are still multiple challenges in the therapy of NSCLC (6). Currently, immune checkpoint inhibitors, such as programmed cell death protein-1 (PD-1) and programmed death receptor ligand-1 (PD-L1) inhibitors, combined with platinum doublet chemotherapy have become the first-line regimen for standard treatment of advanced NSCLC patients negative for driver genes (7). However, there are still advanced NSCLC patients with no responses to the combination therapy or developing resistance to the combination therapy (8). A search for more effective therapy is therefore of an urgent need (6). Current evidence shows that advanced NSCLC patients acquiring resistance to first-line regimen with immunotherapy and chemotherapy have limited second-line treatments with huge unmet clinical needs that remain to be addressed (9). Although targeted therapy drugs remarkable improve the survival among patients with EGFR-mutated NSCLC (10), emergence of drug resistance is widespread (11). Platinum-based chemotherapy plus anti-angiogenic agents has been used as a standard treatment for advanced NSCLC patients that fail to continue to receive targeted therapy following drug resistance; however, the response is limited (12). Therefore, optimizing the first-line regimen for treatment of advanced NSCLC patients negative for driver genes, addressing the challenges of resistance to immunotherapy plus chemotherapy and optimizing the treatment strategies for EGFR-mutated NSCLC patients following resistance to targeted therapy, remain to be addressed in clinical practices (13).

T cells are main tumor-killing immune cells (14); however, the escape mechanisms elicited by multiple types of tumor cells make T cells ineffective to kill tumor cells (15). Recently, the introduction of bispecific T cell engagers (BiTEs) has attracted widespread attention (16). Anti-CD3 BiTEs may simultaneously bind to CD3 on T cell surface and tumor-associated antigens (TAAs) on tumor cell surface, thereby allowing T cell redirecting to tumor cells and facilitating their binding (17). This binding may result in T cell activation and generation of immunological synapses, and activated T cells secrete perforin and other granule enzymes via immunological synapses, which finally leads to tumor cell lysis (18). The functions of BiTEs are not dependent on major histocompatibility complex (MHC)-T cell receptor (TCR)-mediated antigen presentation, and BiTEs is reported to trigger almost all T cells expressing CD3 for highly efficient killing of tumor cells (17). Upon T cell activation, the expression of PD-1 on T cell surface is upregulated, thereby affecting therapeutic efficacy (19). BiTEs combined with anti-PD-1/PD-L1 drugs were reported to restore T-cell effector functions activated by anti-CD3 BiTEs, to trigger a stronger antitumor activity than anti-CD3 BiTEs alone (20).

Previous studies have shown the strong correlation between mutation or overexpression of epidermal growth factor receptor (EGFR) and development of multiple cancers (21). Since EGFR protein expression ranges from 50% to 80% in lung cancer (22), EGFR is therefore accepted as an important target for immunotherapy or targeted therapy of lung cancer (23). As a recombinant anti-EGFR/CD3 bispecific antibody developed by Zhejiang Shimai Pharmaceutical Co., Ltd., SMET12 directs T cell activation to kill EGFR-positive tumor cells through binding to EGFR on tumor cell surface and CD3 receptor on T cell surface (24). SMET12 combined with PD-1 monoclonal antibody toripalimab was reported to exhibit a synergistic anti-tumor effect in patient-derived xenograft (PDX) models, and results from two ongoing clinical trials also showed a preliminary anti-tumor efficacy and high safety (25, 26). Toripalimab is a recombinant, humanized anti-PD1 antibody developed by Shanghai Junshi Bioscience Co., Ltd, and was approved as a first-line treatment for locally advanced or metastatic, non-squamous NSCLC by National Medical Products Administration (NMPA) of China (27). Chemotherapy drugs are effective to kill cancer cells and present a positive immunoregulatory effect to increase anti-tumor immune responses (28).

Current evidence shows that chemotherapy, PD-1 inhibitors and anti-EGFR/CD3 bispecific antibody have their corresponding anti-tumor efficacy. This multi-cohort, prospective, phase 2 clinical trial aimed to investigate the efficacy and safety of SMET12 plus toripalimab and chemotherapy among advanced NSCLC patients tested positive for EGFR protein that were treatment-naïve, with resistance to first-line immune checkpoint inhibitors-containing therapy and harbor *EGFR* mutations and acquire resistance to first-line EGFR-TKIs, and to evaluate the associations of lymphocyte numbers and differentiation pattern with therapeutic efficacy among advanced NSCLC patients through detection of peripheral blood lymphocytes with flow cytometry.

## Methods

### Ethical statement

This trial was approved by the Institutional Ethical Review Committee of Fujian Medical University Cancer Hospital and was registered at ClinicalTrials.gov (NCT06208033). All experimental procedures were performed strictly according to the Declaration of Helsinki, and other international and national ethical laws, guidelines and regulations of biomedical and life studies involving humans. Signed informed consent was obtained from all participants following a detailed description of the purpose and benefits of the trial.

### Study subjects

This is a phase 2, single-arm, investigator-initiated cohort clinical trial performed in Clinical Oncology School of Fujian Medical University, Fujian Cancer Hospital (Fuzhou, China). Eligible subjects were EGFR protein-positive advanced NSCLC patients with immunohistochemistry (IHC) of at least 1+ at ages of 18 years and older, and included three cohorts: Cohort A consisted of treatment-naïve patients, and Cohort B consisted of patients acquiring resistance to first-line immune checkpoint inhibitors-containing therapy, while Cohort C consisted of *EGFR*-mutated patients acquiring resistance to TKIs. The inclusion criteria involved (1) presence of at least one measurable lesion according to the Response Evaluation Criteria in Solid Tumours (RECIST) version 1.1 (29); (2) Eastern Cooperative Oncology Group (ECOG) performance status score of 2 and lower (30); (3) survival for at least 3 months based on investigators’ estimation; and (4) sufficient organ functions. Subjects meeting the following criteria were excluded from the trial: (1) patients with active autoimmune diseases or a medical history of autoimmune diseases that require systemic steroid therapy; (2) patients with double primary malignancies within the past 5 years; (3) patients with active infections requiring systemic therapy; (4) participants in Cohort B developed grade 3 and higher immune-associated adverse reactions upon receiving first-line immune checkpoint inhibitors-containing therapy; and (5) NSCLC participants tested positive for driver genes *EGFR*, *ALK* or *ROS1* in cohort s A and B.

### Treatment regimens

All participants received triple therapy with SMET12, toripalimab and 2 to 4 cycles of chemotherapy, and participants with complete response (CR), partial response (PR) or stable disease (SD) following 2 to 4 cycles of chemotherapy (**Table 1**) were given maintenance therapy with SMET12 plus toripalimab until progressive disease (PD), unacceptable toxicity, death, loss of clinical benefits evaluated by investigators, loss to follow-up, pregnancy, withdrawal of informed consent or termination of the trial. The maintenance therapy was given for no more than 2 years. If chemotherapy and toripalimab were administered at the same day, toripalimab was given at least 30 min following chemotherapy, and SMET12 was intravenously administered on the second day of toripalimab administration during the whole treatment period. In this trial, toripalimab was intravenously administered at a dose of 3 mg/kg, Q2W; SMET was intravenously given at 30 μg on the day following toripalimab administration, Q2W; and chemotherapy was given for 2 to 4 cycles, with three weeks as a cycle (**Table 1**).

**Table 1:** Chemotherapy regimens in three arms.

Cohort	Chemotherapy regimen
A	Non-squamous, non-small-cell lung cancer patients: pemetrexed (500 mg/m^2^, d1) plus carboplatin (AUC = 5; d1, Q3W); lung squamous cell carcinoma patients: albumin-bound paclitaxel (100 mg/m^2^, d1, d8 and d15) plus cisplatin (75 mg/m^2^, d1, Q3W).
B	Patients receiving second-line chemotherapy: docetaxel at a dose of 60 to 75 mg/m^2^, d1, Q3W; those receiving third- and further-line chemotherapy are given regimens by investigators depending on patients’ conditions.
C	Pemetrexed (500 mg/m^2^, d1) plus carboplatin (AUC = 5; d1, Q3W)

### Flow cytometry

In this trial, peripheral blood samples were collected at baseline, cycle 1 day 17 (C1D17) and cycle 3 day 17 (C3D17), 6 months and 12 months, and peripheral blood lymphocytes were measured on a CytoFLEX S flow cytometer (Beckman Coulter, Inc.; Brea, CA, USA) among advanced NSCLC patients pre- and post-treatment to evaluate the associations of lymphocyte numbers and differentiation pattern with therapeutic efficacy, as well as to characterize dynamic changes in these parameters during treatment.

**Figure 1:**
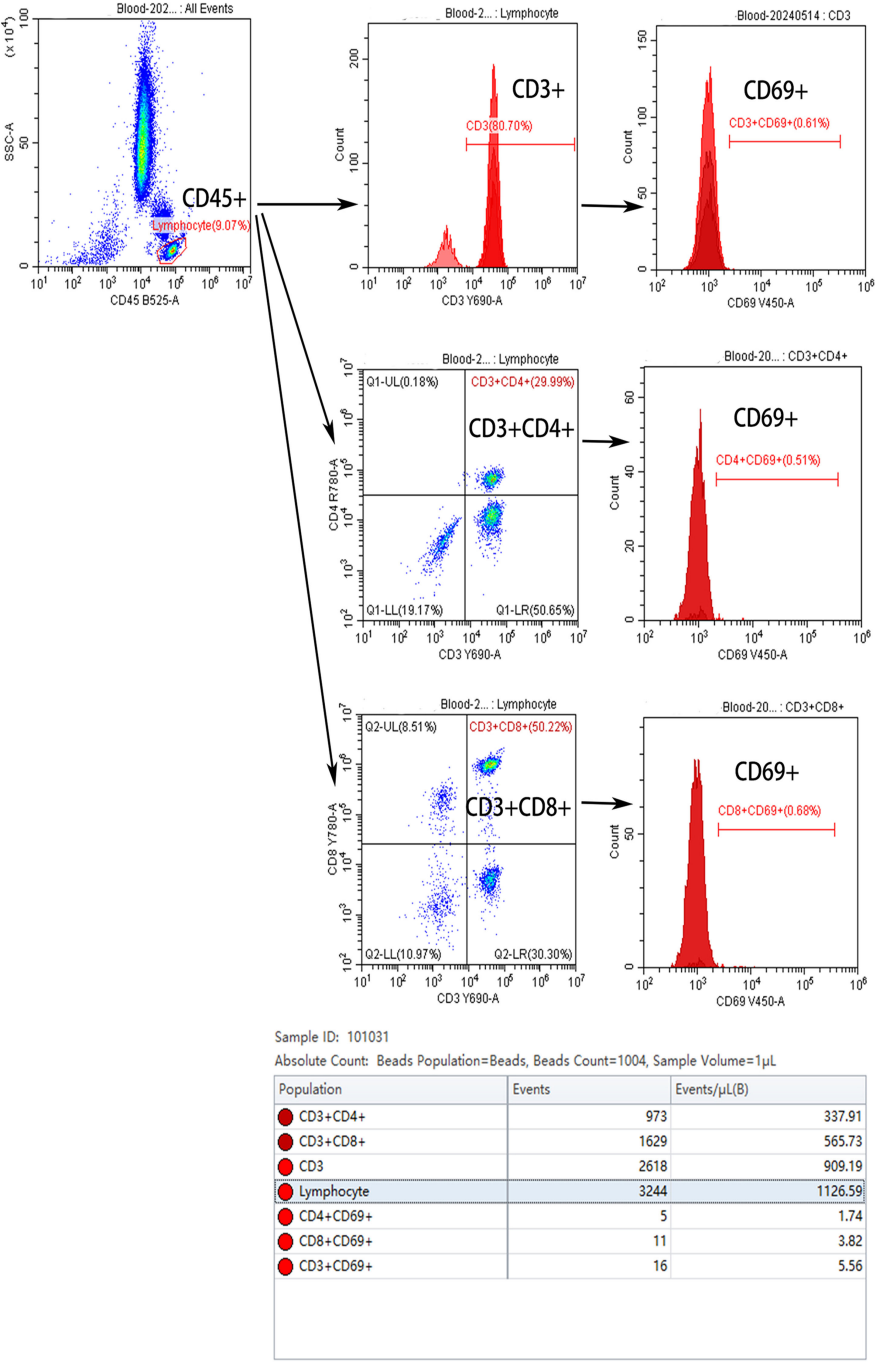
Schematic diagram of flow cytometric analysis for lymphocyte counting.

To measure lymphocyte numbers, 100 μL of peripheral blood samples were transferred into a flow cytometry tube, and 5 to 20 μL of flow cytometry antibodies were added to the tube, followed by a 30-minute incubation at 4°C in the darkness. Subsequently, 500 μL of erythrocyte lysis buffer was added and incubated at room temperature for 5 to 10 minutes. Following complete lysis, 500 μL of phosphate-buffered saline (PBS) was added to stop the reaction. Finally, 100 μL of absolute counting microspheres were added, mixed well, and the sample was ready for flow cytometric analysis. After lysis of red blood cells from peripheral blood, lymphocytes were identified using the CD45-FITC conjugated antibody. Total T lymphocytes (CD3^+^), helper T cells (CD3^+^CD4^+^), and cytotoxic T cells (CD3^+^CD8^+^) were further defined using CD3-PerCP-Cy5.5, CD4-APC-H7 and CD8-PE-Cy7 conjugated antibodies. The activation status of each cell type was assessed by detecting CD69 expression: CD3^+^CD69^+^ for total T cells, CD3^+^CD4^+^CD69^+^ for helper T cells, and CD3^+^CD8^+^CD69^+^ for cytotoxic T cells. The absolute counts of these cell types were calculated using absolute counting beads (**Figure 1**).

**Figure 2:**
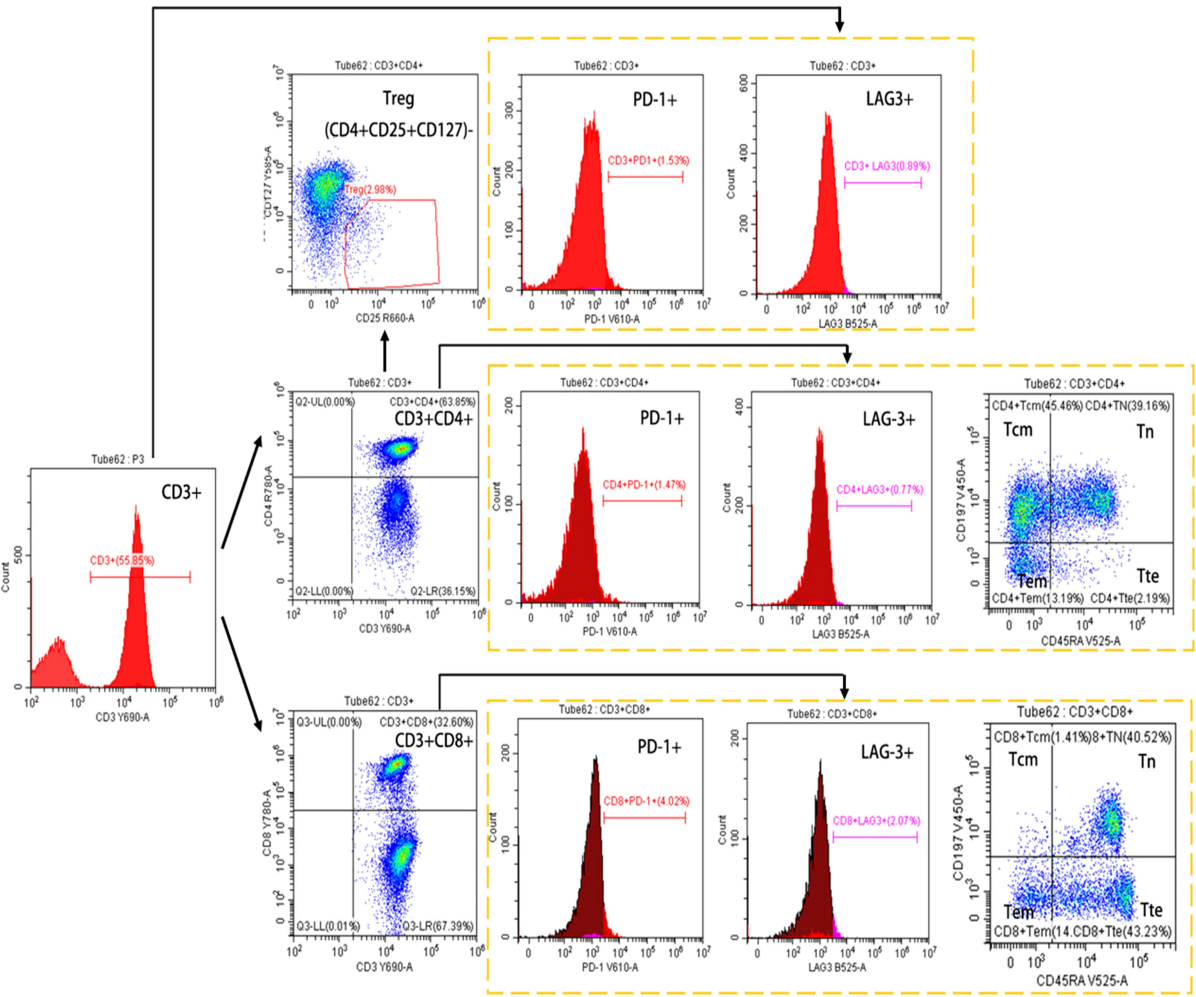
Flow-cytometry gating strategy for the identification of lymphocyte subsets.

To explore the patterns of lymphocyte differentiation, peripheral blood mononuclear cells (PBMCs) were extracted from the patients via a standardized density gradient approach, specifically utilizing Ficoll-Paque PLUS density gradient media (GE Healthcare Biosciences; Piscataway, NJ, USA, catalog number: 17-1440-02). Cells were harvested and subjected to centrifugation at 2,000 r/min for 5 minutes at room temperature. Subsequently, the supernatant was carefully discarded. The harvested cells were then resuspended in 100 μL of PBS supplemented with 2% fetal bovine serum (FBS), following by addition of 5 to 20 μL of the antibody into the cell suspension, and the mixture was incubated at 4°C for 30 minutes. After incubation, cells were washed twice in PBS to remove any unbound substances. Finally, cells were resuspended in 300 μL of PBS and analyzed using flow cytometry. Then, total T lymphocytes (CD3^+^), helper T cells (CD3^+^CD4^+^), and cytotoxic T cells (CD3^+^CD8^+^) were further defined using CD3-PerCP-Cy5.5, CD4-APC-H7 and CD8-PE-Cy7 conjugated antibodies, and the PD-1 and LAG-3 expression was detected in these three cell populations with BV605 anti-human CD279 (PD-1) antibody and anti-CD233 (LAG-3) antibody. In addition, regulatory T cells (Tregs) were identified within CD3^+^CD4^+^ T cells using CD25-APC and CD127-PE conjugated antibodies, and the ratios of naïve T cells (Tn) (CCR7^+^CD45RA^+^), central memory T cells (Tcm) (CCR7^+^CD45RA^-^), effector memory T cells (Tem) (CCR7^-^CD45RA^-^), and terminally differentiated effector T cells (Tte) (CCR7^-^CD45RA^+^) cells in CD3^+^CD4^+^ and CD3^+^CD8^+^ T cells were determined using BV510 anti-human CD45RA and BV421 anti-CD197 (CCR7) antibodies (**Figure 2** is here).

The catalog numbers for all antibodies used in the flow-cytometry experiments were listed below: FITC Mouse Anti-Human CD45 (catalog number: 555482); PerCP-Cy™5.5 Mouse Anti-CD3 (catalog number: 552852); APC-H7 Mouse Anti-CD4 (catalog number: 560158); PE-Cy™7 Mouse Anti-CD8 (catalog number: 566858); APC Mouse Anti-CD25 (catalog number: 555434); PE Mouse Anti-CD127 (catalog number: 557938); BV421 Mouse Anti-CD197 (CCR7) (catalog number: 562555); BV510 Mouse Anti- CD45RA (catalog number: 563031); BV605 Mouse Anti-CD279 (PD-1) (catalog number: 563245); FITC anti-human CD223 (LAG-3) Antibody catalog number: 369308). The antibodies mentioned above were purchased from Becton, Dickinson, and Company (Franklin Lakes, NJ, USA). CD69 Monoclonal Antibody (FN50), eFluor™ 450 (catalog number: 48-0699-42), was purchased from eBioscience, Inc (San Diego, CA, USA).

We quantified serum cytokines at all five protocol-defined time points—baseline, Cycle 1 Day 17 (C1D17), Cycle 3 Day 17 (C3D17), 6 months, and 12 months—using the BD Cytometric Bead Array (CBA) Human Th1/Th2/Th17 Kit (BD Biosciences, cat. #560484). The panel comprised IL-2, IL-4, IL-6, IL-10, TNF-α, IFN-γ, and IL-17A.

### Outcome measures

The primary endpoints of this trial included safety and therapeutic efficacy. The endpoints of therapeutic efficacy included objective response rate (ORR), disease control rate (DCR) and progression-free survival (PFS) evaluated using RECIST 1.1 (29) and Immune-based RECIST (iRECIST) (31), and the exploratory endpoints included the association of lymphocyte numbers and differentiation pattern with clinical benefits. Adverse events and laboratory abnormality were graded according to the United States National Cancer Institute Common Terminology Criteria for Adverse Events 5.0 (32), and during all cycles, each patient’ highest toxicity graded was included in the final toxicity analysis. Baseline imaging assessments were performed within 28 days prior to the first drug administration, disease progression was followed up once every 5 to 7 weeks following the first administration until progressive disease (PD) as revealed by imaging assessments, initiation of new antitumor treatments, death, loss to follow-up, withdrawal of informed consent or termination of the trial.

### Statistical analysis

Based on datasets for analysis of therapeutic efficacy, quantitative tumor measurements were compared between at baseline and during treatment periods and waterfall plots were created, and ORR, DCR and PFS were descriptively analyzed. The confidence interval of percentage was calculated using Clopper-Pearson exact method, and the time endpoints of events were estimated using Kaplan-Meier estimation. The safety and tolerance were analyzed based on datasets for safety analysis, and adverse events during treatment periods, drug-associated adverse events, adverse events of specific concerns and severe adverse events were listed in details. All statistical analyses were performed using the statistical software SPSS 22.0 (SPSS, Inc.; Chicago, IL, USA), and a *P* value of < 0.05 indicated statistical significance.

## Results

### Subject characteristics

**Figure 3:**
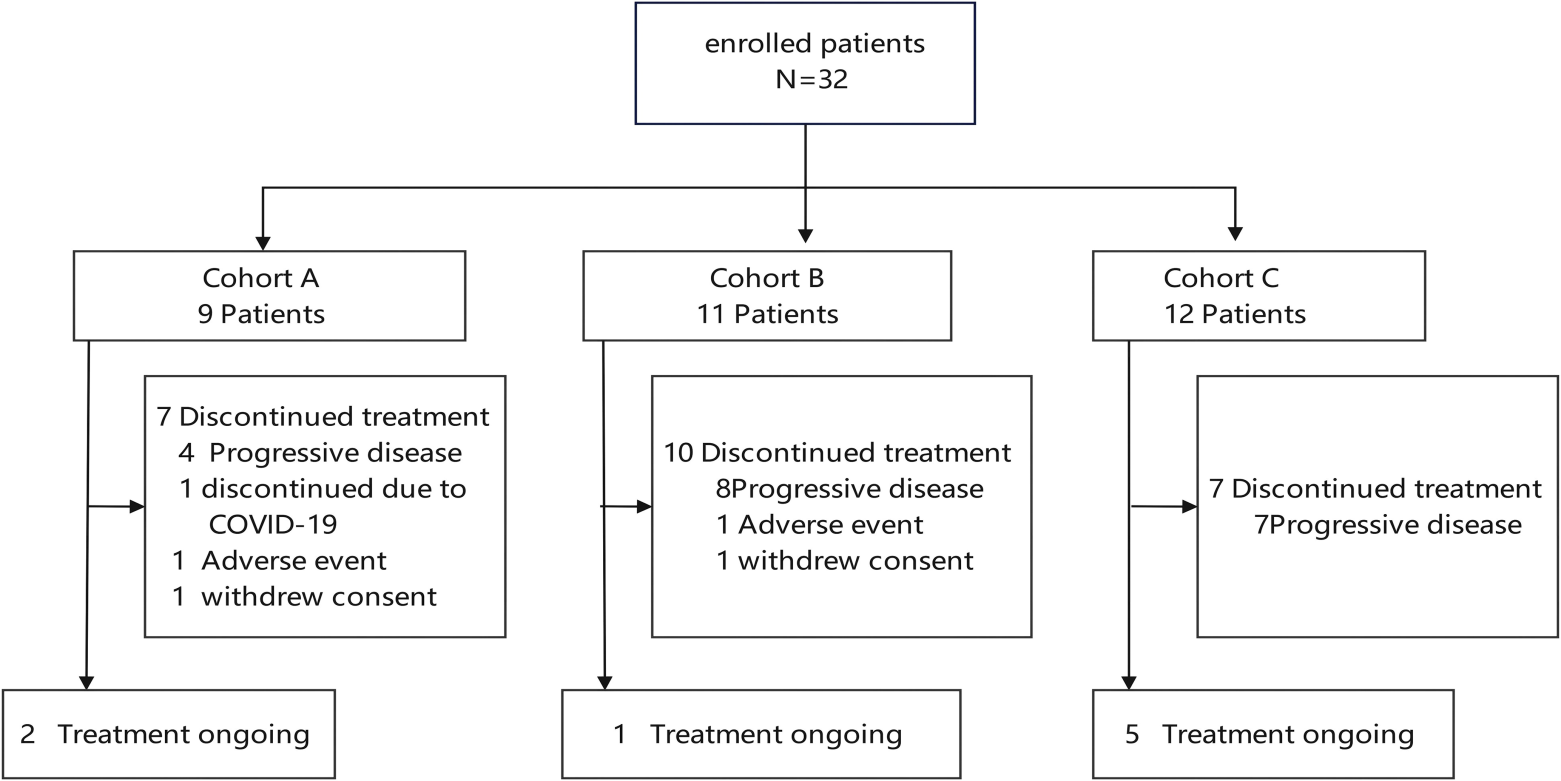
Flowchart of recruitment of study subjects.

A total of 32 patients from Fujian Medical University Cancer Hospital were enrolled in the trial during the period from March 7 to July 16, 2024, and baseline subject characteristics were described in **Table 2**. There were 9 cases in Cohort A, including 6 cases with evaluable tumor responses to treatment and 3 cases with early termination of treatment (only completion of a cycle and without subsequent imaging evaluation), and the reasons for early termination included treatment discontinuation due to COVID-19 (one case), treatment discontinuation due to development of grade 3 immune-related myositis (one case) and withdrawal from the trial (one case). There were 11 cases in Cohort B, including 9 cases with evaluable tumor responses to treatment and 2 cases with early termination of treatment (only completion of a cycle and without subsequent imaging evaluation), and the reasons for early termination included treatment discontinuation due to development of grade 3 immune-related pneumonitis (one case) and withdrawal from the trial (one case). There were 12 cases in Cohort C, and all had evaluable tumor responses to treatment (**Figure 3**). The median follow-up duration was 9 months (range, 4 to 10.5 months) until January 21, 2025. There were still 8 patients under active treatment, including 2 cases in Cohort A, one case in Cohort B and 5 cases in Cohort C.

**Table 2:** Demographic and clinical characteristics of study subjects at baseline.

Characteristic	Cohort A (*n* = 9)	Cohort B (*n* = 11)	Cohort C (*n* = 12)
Median age (years)	66	62	62.5
Gender (%)			
Male	9/9 (100%)	11/11 (100%)	7/12 (58.3%)
Female	0/9 (0)	0/11 (0)	5/12 (41.7%)
History of tobacco use (%)			
Never	3/9 (33.3%)	4/11 (36.4%)	10/12 (83.3%)
Current	4/9 (44.4%)	3/11 (27.3%)	2/12 (16.7%)
Former	2/9 (22.2%)	4/11 (36.4%)	0/12 (0)
ECOG performance-status score			
0	0/9 (0)	0/11 (0)	0/12 (0)
1	9/9 (100%)	11/11 (100%)	12/12 (100%)
2	0/9 (0)	0/11 (0)	0/12 (0)
Tumor histology			
Adenocarcinoma	3/9 (33.3%)	3/11 (27.3%)	11/12 (91.7%)
Squamous carcinoma	4/9 (44.4%)	8/11 (72.7%)	0/12 (0)
Other	2/9 (22.2%)	0/11 (0)	1/12 (8.3%)
Brain metastases			
Yes	2/9 (22.2%)	2/11 (18.2%)	3/12 (25%)
No	7/9 (77.7%)	9/11 (81.8%)	9/12 (75%)
Liver metastases			
Yes	3/9 (33.3%)	0/11 (0)	2/12 (16.7%)
No	6/9 (66.7%)	11/11 (100%)	10/12(83.3%)
Bone metastases			
Yes	1/9 (11.1%)	3/11 (27.3%)	6/12 (50%)
No	8/9 (88.9%)	8/11 (72.7%)	6/12 (50%)
Quantifiable PD-L1 expression			
< 1%	3/9 (33.3%)	4/11 (36.4%)	3/12 (25%)
≥ 1%	2/9 (22.2%)	4/11 (36.4%)	1/12 (8.3%)
Unevaluable	4/9 (44.4%)	3/11 (27.3%)	8/12 (66.7%)
*EGFR* mutation	/	/	12/12 (100%)
*Exon 19* deletion	/	/	6/12 (50%)
Leu858Arg	/	/	6/12 (50%)
Others	/	/	0/12 (0)

### Efficacy

**Figure 4:**
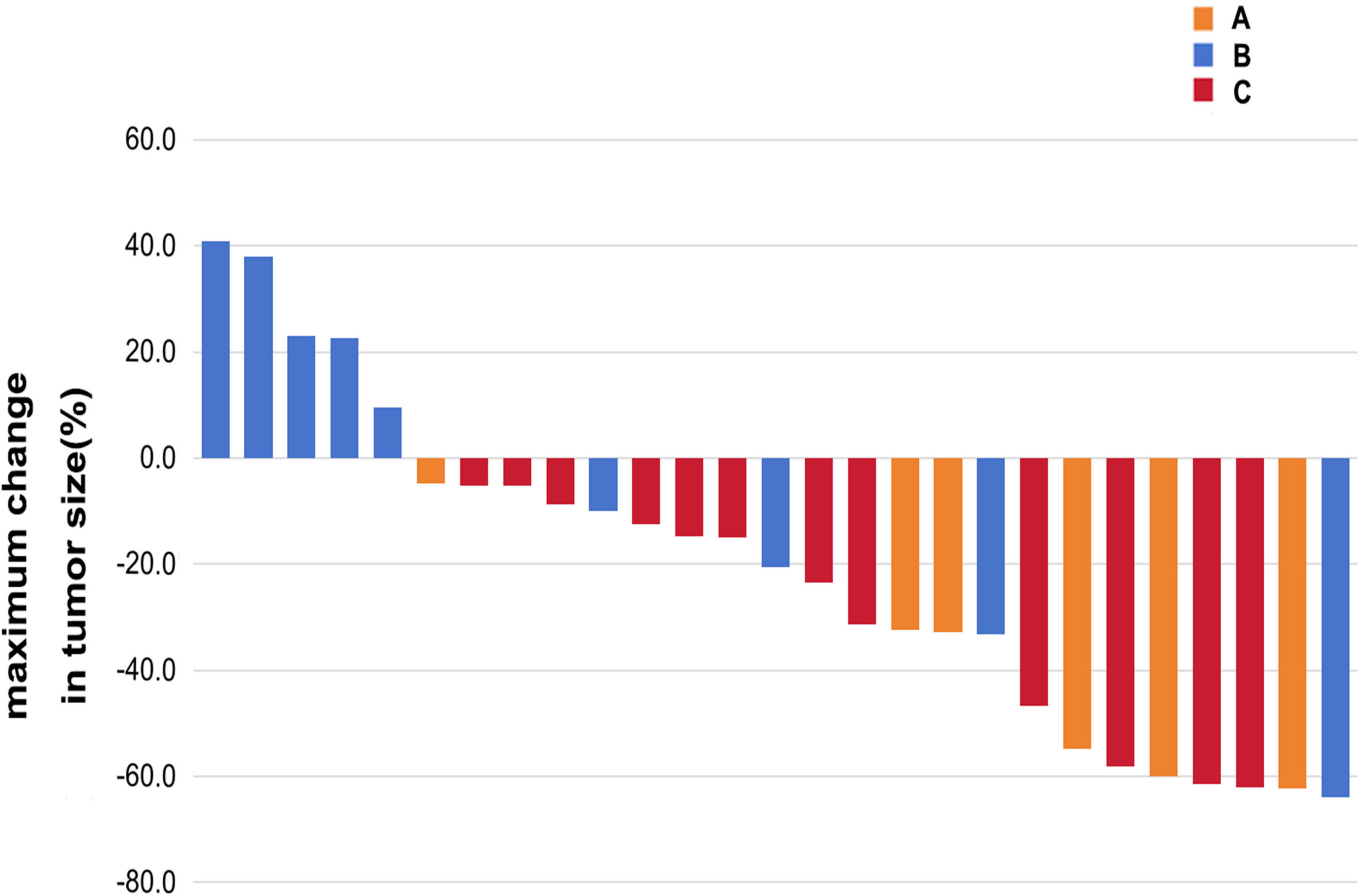
Maximum changes of tumor sizes from baseline by the best overall response, as per response evaluation criteria in solid tumors (RECIST) version 1.1 and iRECIST. Each bar represents the maximum change in the sum of the diameters of the target lesions of an individual patient (*n* = 27). A, B, and C represent Cohort A, Cohort B and Cohort C. Both Cohort A and Cohort C demonstrate tumor size shrinkage.

**Figure 5:**
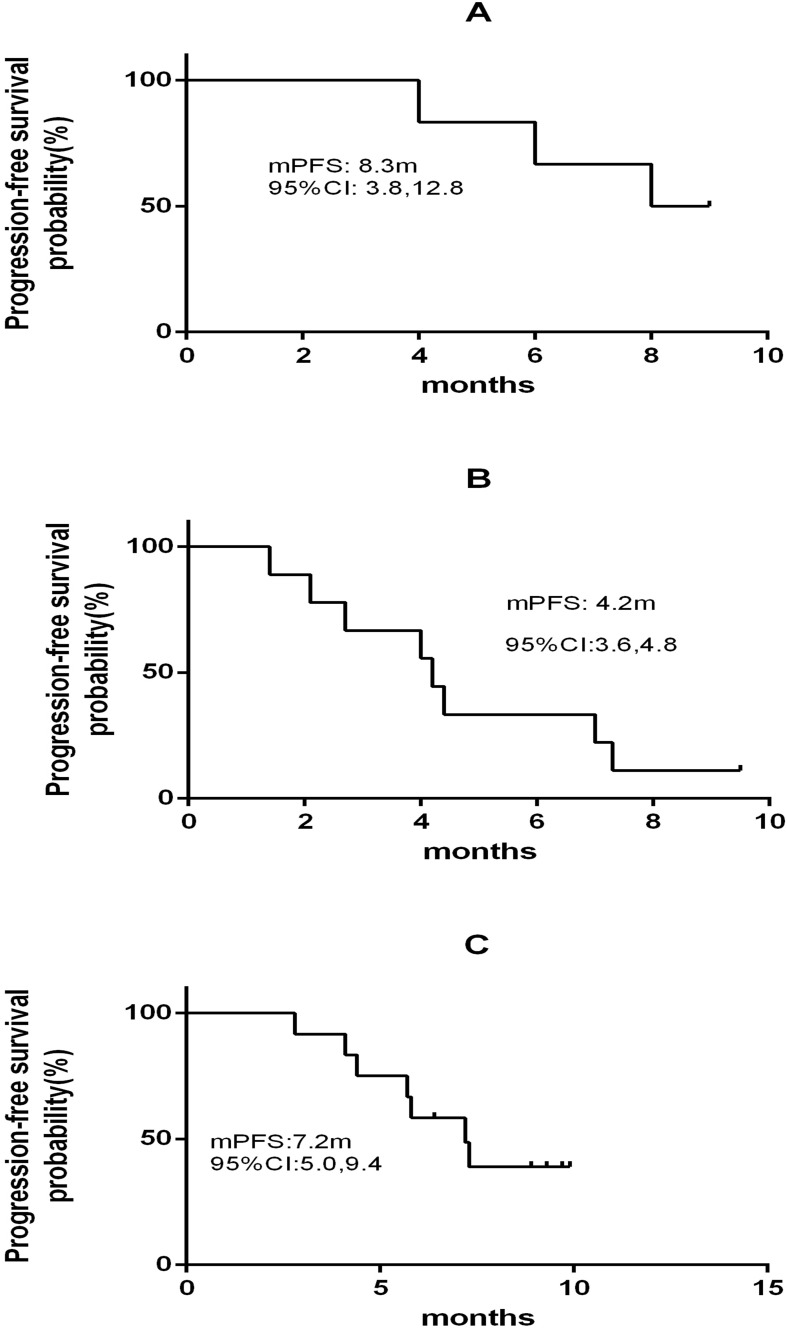
Progression-free survival among subjects in three cohorts. **(A)** Cohort A; **(B)** Cohort B; **(C)** Cohort C.

A total of 24 participants had PD, including 7 cases in Cohort A, 10 cases in Cohort B and 7 cases in Cohort C, and 9 cases died until January 21, 2025, with 3 deaths in each of the three cohorts. However, the overall survival (OS) was unavailable. The ORR, DCR and median PFS were 83.3% (95% *CI*: 35.9% to 99.6%), 100% (95% *CI*: 54.7% to 100%) and 8.3 (95% *CI*: 3.8 to 12.8) months in Cohort A, 22.2% (95% *CI*: 2.8% to 60%), 55.6% (95% *CI*: 21.2% to 86.3%) and 4.2 (95% *CI*: 3.6 to 4.8) months in Cohort B, and 41.7% (95% *CI*: 15.2% to 72.3%), 100% (95% *CI*: 73.5% to 100%) and 7.2 (95% *CI*: 5.0 to 9.4) months in Cohort C (**Table 3**; **Figures 4**, **5**).

**Table 3:** Summary of clinical activity.

Response	Cohort A (*n* = 6)	Cohort B (*n* = 9)	Cohort C (*n* = 12)
Complete response	0	0	0
Partial response	5 (83.3%)	2 (22.2%)	5 (41.7%)
Stable disease	1 (16.7%)	3 (33.3%)	7 (58.3%)
Progressive disease	0	4 (44.4%)	0
Overall response rate (%, 95% *CI*)	83.3 (35.9 to 99.6)	22.2 (2.8 to 60)	41.7 (15.2 to 72.3)
Disease control rate (%, 95% *CI*)	100 (54.7 to 100)	55.6 (21.2 to 86.3)	100 (73.5 to 100)
Progression-free survival (months, 95% *CI*)	8.3 (3.8 to 12.8)	4.2 (3.6 to 4.8)	7.2 (5.0 to 9.4)

The date of data cutoff is January 21, 2025. Objective response is defined as unconfirmed complete response or partial response, as determined by the investigators according to Response Evaluation Criteria in Solid Tumors (RECIST) version 1.1.

### Toxicity

**Table 4** shows adverse events with > 10% incidence in three cohorts. The most common treatment-related adverse events included cytokine release syndrome (53.13%), leukopenia (43.75%), elevated alanine aminotransferase (43.75%), neutropenia (37.5%), constipation (31.25%), elevated aspartate aminotransferase (28.13%), decreased appetite (18.75%), Lung infections (15.63%), hypercholesterolemia (15.63%), fatigue (15.63%), hiccup (12.5%), nausea (12.5%), hypertriglyceridemia (12.5%) and anemia (12.5%). The incidence of grade 3–4 adverse events was 37.5%. These included neutropenia (15.63%), Lung infections (15.63%), anemia (6.25%), immune-related hepatitis (6.25%), immune-related pneumonia (6.25%), leukopenia (3.13%), and hypertriglyceridemia (3.13%). No grade 5 adverse events were observed. Nevertheless, the incidence of grades 3 and 4 TRAEs was numerically higher in cohort B than in cohorts A and C, and notably, the incidence of grades 3 and 4 leukopenia was significantly higher in cohort B than in cohorts A and C. The incidence of CRS was 66.67% in cohort A, 36.36% in cohort B and 58.33% in cohort C. In addition, there were 8 patients (25%) with severe adverse events, including lung infections (15.65%), immune-related pneumonitis (9.38%), immune-related hepatitis (6.25%), atrial fibrillation (3.13%), COVID-19 (3.13%), generalized weakness (3.13%), and neutropenia (3.13%). Immune-related adverse events included immune-related pneumonia (9.38%), immune-related hepatitis (6.25%), elevated thyroid stimulating hormone (3.13%) and hypothyroidism (3.13%) (**Table 4**).

**Table 4:** Incidence of treatment-related adverse events.

Adverse events	Cohort A (*n* = 9)	Cohort B (*n* = 11)	Cohort C (*n* = 12)	ALL (*n* = 32)
Treatment-related adverse events				
Any grade	9 (100)	11 (100)	12 (100)	32 (100)
Grade 3 to 4	3 (33.33)	6 (54.55)	3 (25)	12 (37.5)
Grade 5	0	0	0	0
Events of interest during treatment period				
Cytokine-release syndrome				
Any grade	6 (66.67)	4 (36.36)	7 (58.33)	17 (53.13)
Grade 3 to 4	0	0	0	0
Immune-related adverse events				
Pneumonitis				
Any grade	1 (11.11)	1 (9.09)	1 (8.33)	3 (9.38)
Grade 3 to 4	0	1 (9.09)	1 (8.33)	2 (6.25)
Hepatitis				
Any grade	1 (11.11)	0	1 (8.33)	2 (6.25)
Grade 3 to 4	1 (11.11)	0	1 (8.33)	2 (6.25)
Hypothyroidism				
Any grade	1 (11.11)	0	0	1 (3.13)
Grade 3 to 4	0	0	0	0
Elevated thyroid-stimulating hormone				
Any grade	0	0	1 (8.33)	1 (3.13)
Grade 3 to 4	0	0	0	0
Treatment-related adverse events with an incidence rate of 10% and higher				
Cytokine-release syndrome				
Any grade	6 (66.67)	4 (36.36)	7 (58.33)	17 (53.13)
Grade 3 to 4	0	0	0	0
Leukopenia				
Any grade	3 (33.33)	5 (45.45)	6 (50.00)	14 (43.75)
Grade 3 to 4	0	1 (9.09)	0	1 (3.13)
Elevated alanine aminotransferase				
Any grade	7 (77.78)	1 (9.09)	6 (50.00)	14 (43.75)
Grade 3 to 4	0	0	0	0
Neutropenia				
Any grade	2 (22.22)	6 (54.55)	4 (33.33)	12 (37.5)
Grade 3 to 4	1 (11.11)	4 (36.36)	0	5 (15.63)
Constipation				
Any grade	4 (44.44)	1 (9.09)	5 (41.67)	10 (31.25)
Grade 3 to 4	0	0	0	0
Elevated aspartate aminotransferase				
Any grade	5 (55.56)	1 (9.09)	3 (25.00)	9 (28.13)
Grade 3 to 4	0	0	0	0
Decreased appetite				
Any grade	1 (11.11)	2 (18.18)	3 (25.00)	6 (18.75)
Grade 3 to 4	0	0	0	0
Lung infection				
Any grade	2 (22.22)	2 (18.18)	1 (8.33)	5 (15.63)
Grade 3 to 4	2 (22.22)	2 (18.18)	1 (8.33)	5 (15.63)
Hypercholesterolemia				
Any grade	1 (11.11)	1 (9.09)	3 (25.00)	5 (15.63)
Grade 3 to 4	0	0	0	0
Fatigue				
Any grade	1 (11.11)	1 (9.09)	3 (25.00)	5 (15.63)
Grade 3 to 4	0	0	0	0
Hiccup				
Any grade	2 (22.22)	2 (18.18)	0	4 (12.5)
Grade 3 to 4	0	0	0	0
Nausea				
Any grade	0	1 (9.09)	3 (25.00)	4 (12.5)
Grade 3 to 4	0	0	0	0
Hypertriglyceridemia				
Any grade	1 (11.11)	1 (9.09)	2 (16.67)	4 (12.5)
Grade 3 to 4	0	0	1 (8.33)	1 (3.13)
Anemia				
Any grade	1 (11.11)	1 (9.09)	2 (16.67)	4 (12.5)
Grade 3 to 4	0	1 (9.09)	1 (8.33)	2 (6.25)
Serious adverse events	2 (22.22)	4 (36.36)	2 (16.67)	8 (25)

The safety analyses include all patients who received at least one dose of therapy. Adverse events are graded according the Common Terminology Criteria for Adverse Events (CTCAE) version 5.0. The severity of cytokine-release syndrome (CRS) is typically assessed using the American Society for Transplantation and Cell Therapy (ASTCT) consensus grading system (grades 1 to 4).

### Exploratory analysis of peripheral T lymphocyte subsets

**Figure 6:**
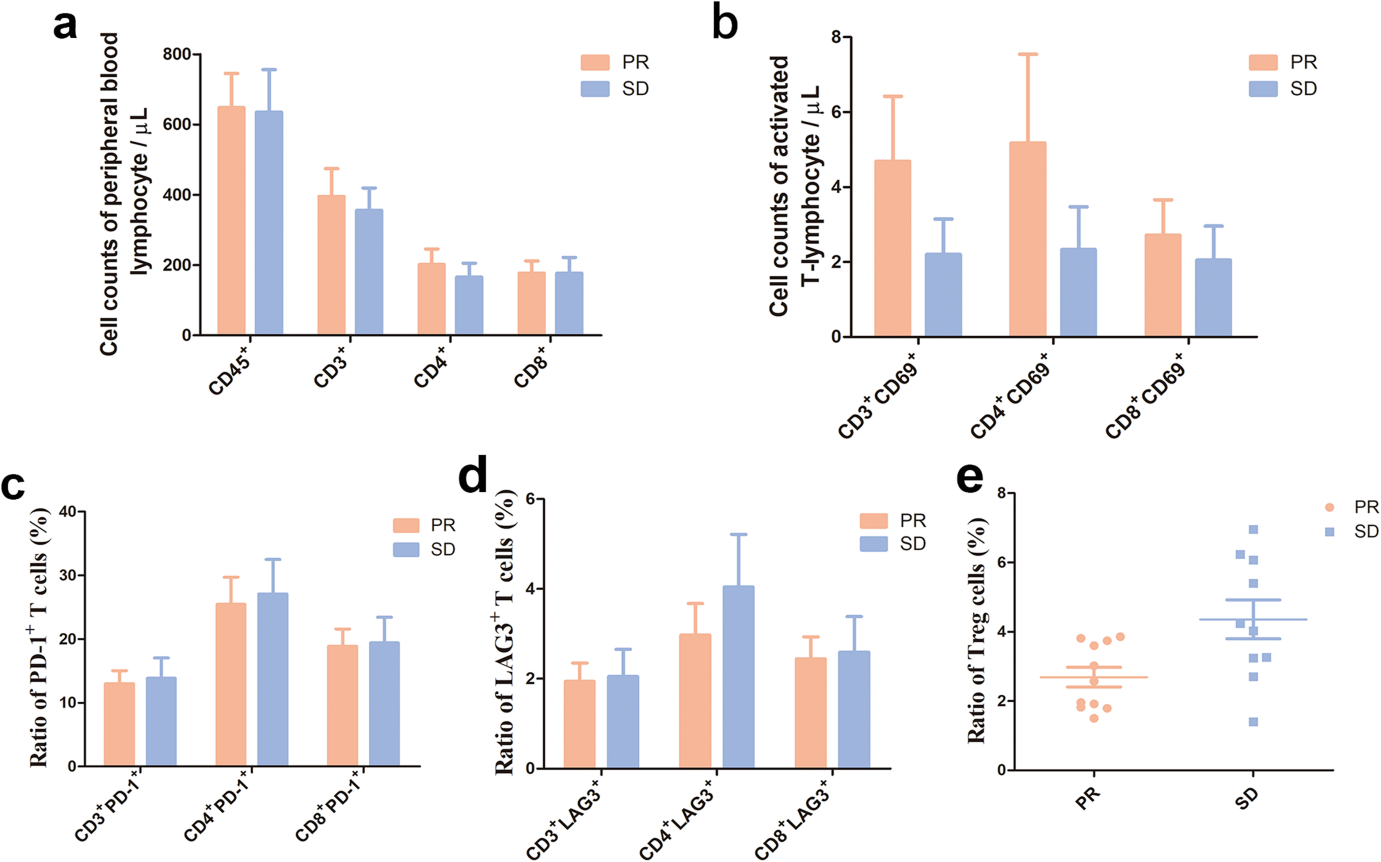
Peripheral blood lymphocyte quantification and subtype profiling in NSCLC patients with partial response or stable disease at baseline. **(a)**, Peripheral blood lymphocyte quantification; **(b)**, Quantification of activated T-lymphocytes; **(c)**, Proportions of PD-1^+^ T cells in CD3^+^, CD4^+^ and CD8^+^T cells; **(d)**, Proportions of LAG3^+^ T in CD3^+^, CD4^+^ and CD8^+^T cells; **(e)**, Proportions of Treg cells: proportion of Treg (CD3^+^CD4^+^CD25^+^CD127^-^) cells in CD4^+^T cells. **(a)** (PR group: n = 9; SD group: n = 8); **(b)** (PR group: n = 6; SD group: n = 4); **(c–e)** (PR group: n = 11; SD group: n=10). The baseline lymphocyte count and activated T-cell count are numerically greater in NSCLC patients with partial response than those with stable disease (*P* > 0.05) **(a, b)**, and the PD-1 positive T-cell ratio and LAG3-positive T-cell ratio are numerically higher among NSCLC patients with stable disease than those with partial response (*P* > 0.05) **(c, d)**. The Treg cell ratio is significantly higher among NSCLC patients with stable disease than those with partial response (*P* = 0.03) **(e)**.

**Figure 7:**
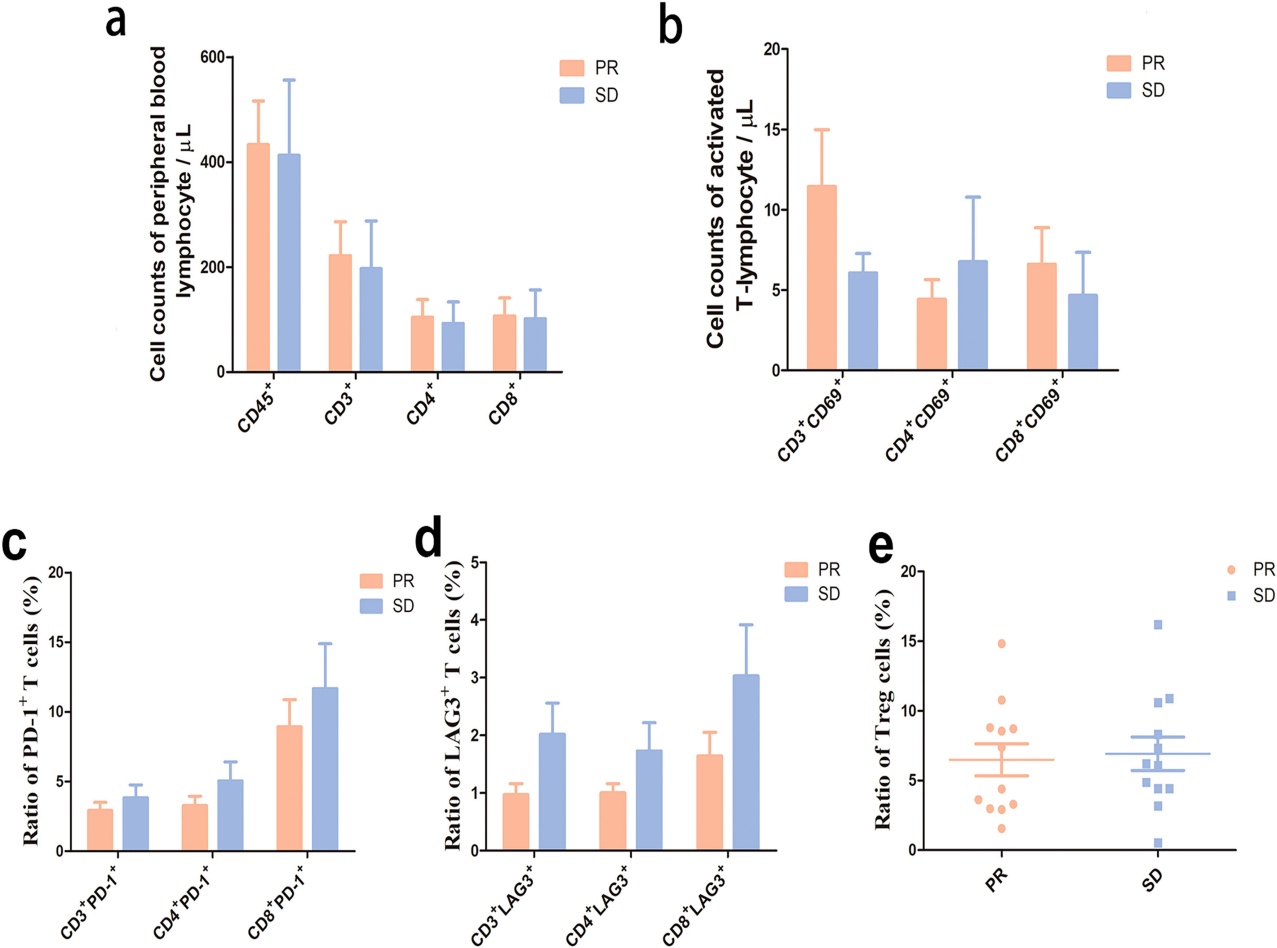
Peripheral blood lymphocyte quantification and subtype profiling among NSCLC patients with partial response or stable disease at C3D17. **(a)** Peripheral blood lymphocyte quantification; **(b)**, Quantification of activated T-lymphocytes; **(c)**, Proportions of PD-1^+^ T cells in CD3^+^ CD4^+^ and CD8^+^ T cells; **(d)**, Proportions of LAG3^+^ T cells in CD3^+^, CD4+ and CD8^+^ T cells; **(e)**, Proportions of Treg cells: proportion of Treg (CD3^+^CD4^+^CD25^+^CD127^-^) cells in CD4^+^ T cells. PR group: n = 12; SD group: n = 12. Peripheral blood lymphocyte counts were numerically greater in NSCLC patients with partial response than in those with stable disease at C3D17 (*P* > 0.05) **(a)**, and the absolute counts of activated T lymphocyte subsets (including CD3^+^CD69^+^ and CD8^+^CD69^+^ T lymphocytes) are numerically greater in NSCLC patients with partial response than those with stable disease (*P* > 0.05) **(b)**. The ratios of PD-1^+^ T cells and LAG3^+^ T cells are numerically lower in NSCLC patients with partial response than those with stable disease (*P* > 0.05) **(c, d)**, and the ratio of Treg in the peripheral blood is numerically lower in NSCLC patients with partial response than those with stable disease (*P* > 0.05) **(e)**.

As of January 21, 2025, peripheral blood samples were collected from advanced NSCLC patients at 93 case-times, including 27 patients with blood collection three times and 15 patients with blood collection four times. Because of restricted follow-up period, no blood samples were collected at 12 months post-treatment. Among the three cohorts, partial response (PR) was observed in 12 patients, stable disease (SD) in 12 patients, and progressive disease (PD) in 3 patients. As peripheral T lymphocyte analysis was not performed among 2 out of the three 3 cases with PD, comparative analyses of lymphocyte counts and subsets were only performed between patients with PR and SD (**Table 5**). Flow cytometry showed numerically higher lymphocyte counts and numbers of activated T cells, and lower proportions of PD-1^+^ T cells and LAG-3^+^ T cells among patients with PR than among those with SD at baseline and during the whole treatment period (*P* > 0.05). In addition, the proportion of Treg cells was significantly lower among patients with PR than among those with SD (*P* = 0.03) (**Figures 6**, **7**).

**Table 5:** Peripheral blood lymphocyte counts and subtype profiling in patients with partial response and stable disease.

	Baseline							C1D17							C3D17							6 months						
Absolute lymphocyte count	PR			SD				PR			SD				PR			SD				PR			SD			
	Mean	SEM	N	Mean	SEM	N	P	Mean	SEM	N	Mean	SEM	N	P	Mean	SEM	N	Mean	SEM	N	P	Mean	SEM	N	Mean	SEM	N	P
CD45^+^	647.54	97.36	9	635.22	120.90	8	1.000	538.87	112.07	11	416.81	81.47	11	0.599	432.98	83.33	12	412.74	143.07	12	0.544	895.61	124.99	8	672.43	211.80	7	0.152
CD3^+^	395.21	79.45	9	355.79	63.88	8	0.815	291.04	63.70	11	219.97	58.17	11	0.237	221.49	64.61	12	196.97	90.60	12	0.403	536.75	77.14	8	332.31	89.45	7	0.189
CD4^+^	201.36	44.55	9	165.89	39.19	8	0.673	173.87	44.78	11	95.86	15.99	11	0.149	104.11	33.68	12	92.65	40.59	12	0.830	249.42	47.14	8	125.39	29.84	7	0.097
CD8^+^	177.16	34.59	9	176.43	45.58	8	0.743	110.47	19.91	11	107.78	45.68	11	0.212	106.80	34.09	12	101.41	55.05	12	0.260	258.79	45.84	8	189.40	61.88	7	0.397
CD3^+^CD69^+^	4.69	1.74	6	2.20	0.95	4	0.610	9.78	3.11	9	9.89	4.62	9	1.000	11.44	3.55	12	6.07	1.21	12	0.751	12.03	4.12	8	5.03	1.63	7	0.336
CD3^+^CD4^+^CD69^+^	5.18	2.37	6	2.33	1.14	4	0.476	6.39	1.05	9	7.91	2.37	9	0.796	4.42	1.22	12	6.76	4.02	12	0.977	1.20	0.46	8	1.18	0.31	7	0.862
CD3^+^CD8^+^CD69^+^	2.71	0.95	6	2.05	0.91	4	0.914	5.75	1.41	9	7.15	3.08	9	0.436	6.60	2.28	12	4.66	2.69	12	0.644	9.03	3.37	8	2.55	0.80	7	0.189
Lymphocyte ratio																												
CD3^+^PD1^+^ cells in CD3^+^ T cells (%)	12.99	2.05	11	13.84	3.22	10	0.860	2.16	0.58	11	2.71	1.31	11	0.555	2.93	0.58	12	3.85	0.91	12	0.544	6.47	2.15	8	3.57	1.44	7	0.613
CD3^+^CD4^+^PD1^+^ cells in CD4^+^ T cells (%)	25.44	4.28	11	27.10	5.39	10	0.916	3.18	1.01	11	2.99	0.34	11	0.108	3.27	0.68	12	5.05	1.36	12	0.286	2.65	0.93	8	2.61	0.89	7	0.955
CD3^+^CD8^+^PD1^+^, cells in CD8^+^ T cells (%)	18.89	2.71	11	19.39	4.04	10	0.699	7.62	2.09	11	8.89	3.48	11	0.948	8.93	1.96	12	11.68	3.21	12	0.708	16.37	4.55	8	10.78	3.37	7	0.397
CD3^+^LAG3^+^ cells in T cells (%)	1.94	0.41	11	2.05	0.61	10	0.944	1.64	0.44	11	2.89	0.46	11	0.026*	0.97	0.19	12	2.02	0.54	12	0.157	0.96	0.29	8	0.83	0.26	7	0.955
CD3^+^CD4^+^LAG3^+^ cells in CD4^+^ T cells (%)	2.97	0.70	11	4.05	1.17	10	0.647	1.82	0.45	11	3.07	0.63	11	0.115	1.00	0.16	12	1.73	0.49	12	0.403	1.15	0.40	8	0.92	0.25	7	0.862
CD3^+^CD8^+^LAG3^+^ cells in CD8^+^ T cells (%)	2.45	0.49	11	2.60	0.79	10	0.725	2.56	0.65	11	4.59	0.73	11	0.022*	1.64	0.41	12	3.03	0.89	12	0.204	1.20	0.34	8	1.04	0.32	7	0.779
Treg cells in CD4^+^ T cells (%)	2.69	0.28	11	4.36	0.56	10	0.031*	3.32	1.13	11	7.87	1.25	11	0.011*	6.48	1.15	12	6.92	1.20	12	0.708	3.29	0.53	8	4.86	1.14	7	0.336

Mean represents the arithmetic mean, SEM indicates the standard error of the mean, and N denotes the number of patients. Due to the limited follow-up period, we failed to collect blood samples from patients at the 12-month time point.

* indicates signficant difference.

### Association between therapeutic efficacy and lymphocyte counts/subtypes

**Figure 8:**
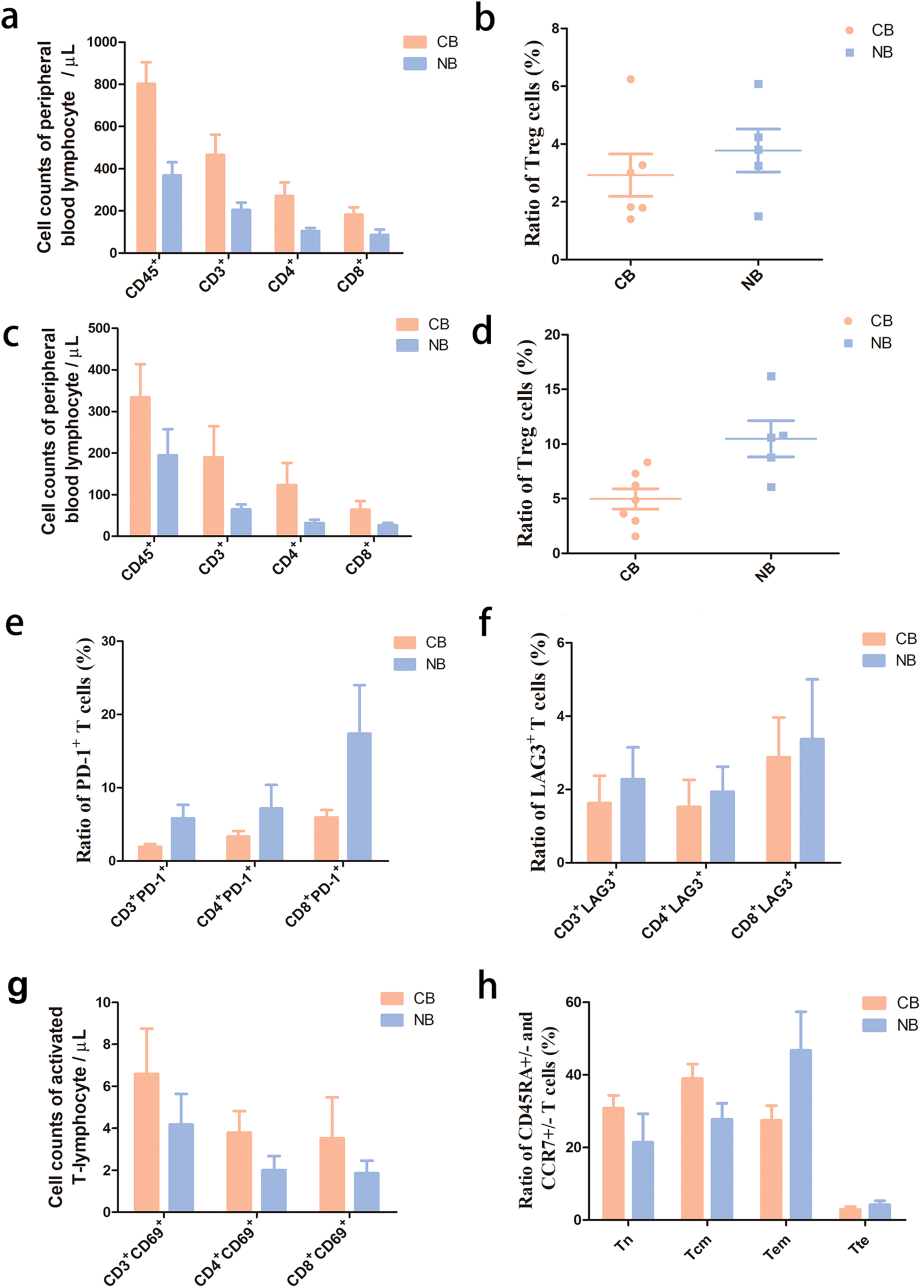
Peripheral blood lymphocyte quantification and subtype profiling among Cohort C patients with or without clinical benefits at baseline and C3D17. **(a)** Peripheral blood lymphocyte quantification at baseline;**(b)**, Proportions of Treg cells: proportion of Treg (CD3^+^CD4^+^CD25^+^CD127^-^) cells in CD4^+^ T cells at baseline; **(c)**, Peripheral blood lymphocyte quantification at C3D17; **(d)**, Proportions of Treg cells at C3D17; **(e)**, Proportions of PD-1^+^ T cells in CD3^+^ CD4^+^ and CD8^+^ T cells at C3D17; **(f)** Proportions of LAG3^+^ T cells in CD3^+^, CD4^+^ and CD8^+^ T cells at C3D17; **(g)**, Quantification of activated T-lymphocytes at C3D17; **(h)**, Ratios of Tn, Tcm Tem and Tte at C3D17. CB, clinical benefits (PFS of 6 months and longer); NB, no clinical benefits (PFS of < 6 months). Tn, naïve T cell; Tcm, central memory T cell; Tem, effector memory T cell; Tte, terminally differentiated effector T cell. **(a)** (CB group: n = 5; NB group: n = 5); **(b)** (CB group: n = 6; NB group: n = 5); **(c–h)** (CB group: n = 7; NB group: n = 5). Flow cytometry detected numerically higher numbers of peripheral blood lymphocyte (CD45^+^, CD3^+^, CD3^+^ CD4^+^ and CD3^+^ CD8^+^ T cells) counts and a numerically lower proportion of Treg cells among patients with CB than among those with NB at baseline (*P* > 0.05), and numerically higher numbers of peripheral blood lymphocyte counts and CD69^+^ T cells, and numerically lower proportions of PD-1^+^ T cells and LAG-3^+^ T cells among patients with CB than among those with NB at C3D17. Numerically higher proportions of CD4^+^ Tcm and Tn cells and lower proportions of CD4^+^ Tem and Tte cells were detected among patients with CB than among those with NB at C3D17.

To examine the association between the therapeutic efficacy and peripheral blood lymphocyte subtypes, PFS of 6 months or longer in Cohort C was defined as clinical benefit (CB), while <6 months was defined as no clinical benefit (NB). Flow cytometry detected numerically higher numbers of peripheral blood lymphocyte (CD45^+^, CD3^+^, CD3^+^ CD4^+^ and CD3^+^ CD8^+^ T cells) counts and a numerically lower proportion of Treg cells among patients with CB than among those with NB at baseline (*P* > 0.05), and numerically higher numbers of peripheral blood lymphocyte counts and CD69^+^ T cells, and numerically lower proportions of Treg cells, PD-1^+^ T cells and LAG-3^+^ T cells among patients with CB than among those with NB at C3D17 (*P* > 0.05). In addition, numerically higher proportions of CD4^+^ Tcm and Tn cells and lower proportions of CD4^+^ Tem and Tte cells were detected among patients with CB than among those with NB at C3D17 (*P* > 0.05) (**Table 6**, **Figure 8**).

**Table 6:** Ratios of memory T cells among NSCLC patients with and without clinical benefits at C3D17.

Lymphocyte ratio	Clinical benefit			No clinical benefit			*P*
	Mean	SEM	N	Mean	SEM	N	
Tn (CD3^+^CD4^+^CCR7^+^CD45RA^+^)	30.78	3.51	7	21.36	7.88	5	0.343
Tcm (CD3^+^CD4^+^CCR7^+^CD45RA^-^)	38.94	4.05	7	27.76	4.35	5	0.073
Tem (CD3^+^CD4^+^CCR7^-^CD45RA^-^)	27.42	4.07	7	46.69	10.64	5	0.149
Tte (CD3^+^CD4^+^CCR7^-^CD45RA^+^)	2.86	0.84	7	4.18	1.12	5	0.268

**Figure 9:**
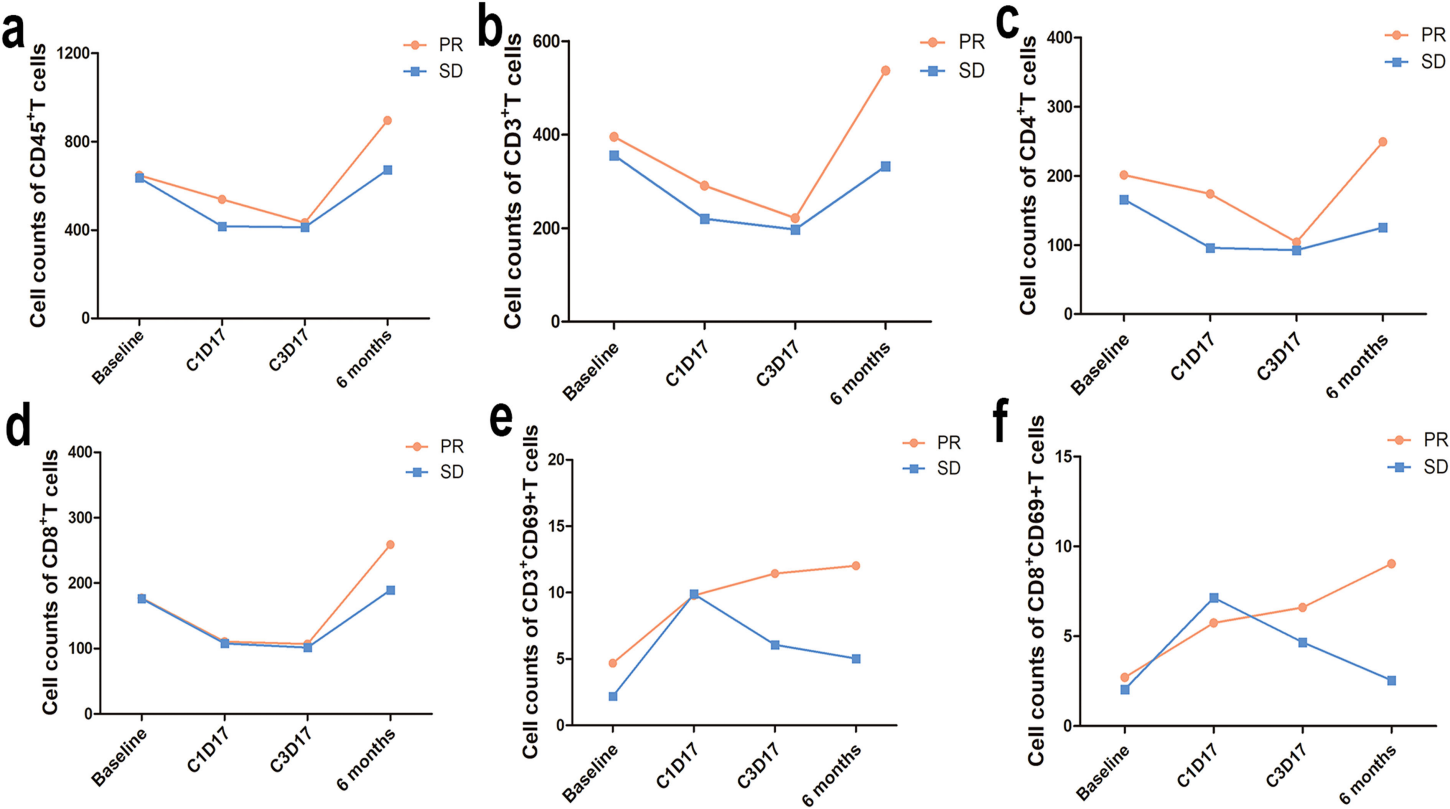
Trends in numbers of CD45^+^, CD3^+^, CD4^+^ and CD8^+^ lymphocyte subsets and activated T-cells counts. **(a–d)**, Changes in the quantities of CD45^+^, CD3^+^, CD4^+^ and CD8^+^ T cells at baseline, C1D17, C3D17, and 6 months post-treatment. **(a–d)** (PR group: n = 9 at baseline, n = 11 at C1D17, n = 12 at C3D17, n = 8 at 6 months; SD group: n = 8 at bassline, n = 11 at C1D17, n = 12 at C3D17, n = 7 at 6 months); **(e, f)** (PR group: n = 4 at baseline, n = 9 at C1D17, n = 12 at C3D17, n = 8 at 6 months; SD group: n = 3 at baseline, n = 9 at C1D17, n = 12 at C3D17, n = 7 at 6 months). Regardless of whether NSCLC patients achieved partial response or stable disease, the counts of all immune-cell subsets tended to decline during the treatment. lymphocyte counts began to rise 6 months post-treatment (12 weeks after chemotherapy), and the increase curve is more obvious among NSCLC patients with partial response; **(e, f)**, Changes in the quantity of CD3^+^CD69^+^ T lymphocytes and CD8^+^CD69^+^ T lymphocytes during the treatment. Activated T-cell counts continued to rise among patients with partial response, whereas in those with stable disease, they increase at C1D17 before gradually declining.

Next, flow cytometry detected a tendency towards a decline in numbers of peripheral blood lymphocyte subsets (CD45^+^, CD3^+^, CD3^+^ CD4^+^ and CD3^+^ CD8^+^ T cells) counts during the treatment period, and the numbers of all lymphocyte subsets started to restore at 6 months (12 weeks post-treatment), with a more remarkable recovery seen among patients with PR than among those with SD. In addition, the numbers of activated T cells (CD3^+^ CD69^+^ and CD8^+^ CD69^+^ T lymphocytes) continued to rise among patients with PR during the treatment period, while they transiently rose among patients with SD at C1D17, and then gradually declined (**Figure 9**).

### Peripheral blood lymphocyte counts and subtype profiling in three cohorts at baseline

**Figure 10:**
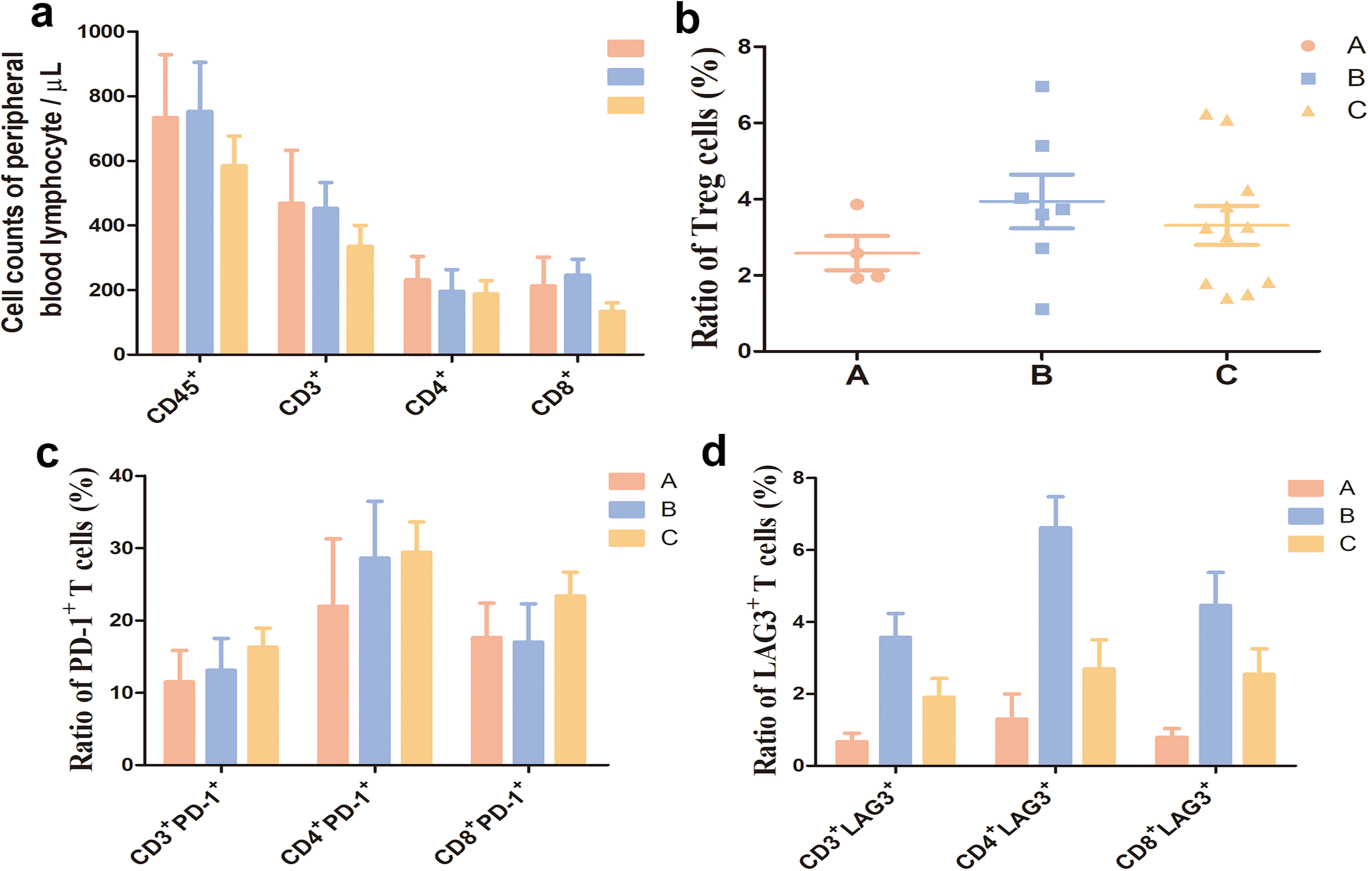
Peripheral blood lymphocyte counts and subtype profiling in three cohorts at baseline. **(a)**, Peripheral blood lymphocyte quantification; **(b)**, Treg cell ratio at baseline; **(c)**, Proportions of PD-1^+^ T cells in CD3^+^ CD4^+^ and CD8^+^ T cells at baseline; **(d)**, Proportions of LAG3^+^ T cells in CD3^+^ CD4^+^ and CD8^+^ T cells at baseline. A, B, and C represent cohorts A, B and C **(a)** (Cohort A: *n* = 3; Cohort B: *n* = 5; Cohort C: *n* = 10); **(b–d)** (Cohort A: *n* = 4; Cohort B: *n* = 7; Cohort C: *n* = 11). Flow cytometry revealed that across the three cohorts A, B, and C, cohort A exhibits the lowest proportions of Treg (P > 0.05), PD-1^+^ T cells (P > 0.05), and LAG-3^+^ T cells (P = 0.006) **(b–d)** at baseline. Cohort B displays the highest proportions of Treg cells (P > 0.05) and LAG-3^+^ T cells (P = 0.006) **(c, d)**. Patients in cohort C have the lowest peripheral blood lymphocyte counts (CD45^+^, CD3^+^, CD3^+^CD4^+^, and CD3^+^CD8^+^ T cells) (P > 0.05) and the highest proportion of PD-1^+^ T cells (P > 0.05) at baseline.

Flow cytometry was performed to detect peripheral blood lymphocyte subset counts, and proportions of Treg cells, PD-1^+^ T cells and LAG-3^+^ T cells to unravel the immune status in the three cohorts at baseline. The lowest proportions of Treg cells, PD-1^+^ T cells and LAG-3^+^ T cells were detected in cohort A. (**Figures 10a–d**), and the highest proportions of Treg cells, and LAG-3^+^ T cells were seen in cohort B (**Figures 10c, d**). In addition, the lowest counts of peripheral blood lymphocyte subsets (CD45^+^, CD3^+^, CD3^+^CD4^+^and CD3^+^CD8^+^T cells) were recorded in cohort C; however, the highest proportion of PD-1^+^ T cells was seen in cohort C. Taken together, the lowest degree of immune exhaustion was detected in cohort A, followed by in cohort C, and the highest was seen in cohort B.

We quantified serum cytokines at all five protocol-defined time points—baseline (n=27), Cycle 1 Day 17 (C1D17) (n=25), Cycle 3 Day 17 (C3D17) (n=26), 6 months (n=25), and 12 months (n=8). The panel comprised IL-2, IL-4, IL-6, IL-10, TNF-α, IFN-γ, and IL-17A. However, most analytes either remained within the normal laboratory range or were below the kit’s lower limit of detection (LLOD), so these results were not included in the original figures. Raw CBA data are supplied in the **Supplementary File**.

## Discussion

To the best of our knowledge, this is the first clinical trial to report the efficacy and safety of an EGFR/CD3 bispecific antibody combined with PD-1 antibody and chemotherapy among NSCLC. SMET12 is a bispecific T-cell antibody targeting EGFR/CD3 (24). Results from this phase 2 clinical trial showed that the triple regimen consisting of SMET12, toripalimab and chemotherapy, showed manageable safety and anti-tumor action among advanced NSCLC patients positive for EGFR protein expression. Notably, this triple regimen achieved an impressive clinical efficacy among EGFR-mutated NSCLC patients with PD following TKIs treatment.

Immune checkpoint inhibitors in combination with platinum-based doublet chemotherapy have been recommended as the first-line standard regimen for treatment of driver gene-negative advanced NSCLC patients (33). Results from multiple phase 3 clinical trials showed that immune checkpoint inhibitors in combination with platinum-based doublet chemotherapy resulted in median PFS of 7.0 to 11.3 months and ORR of 45% to 75% among advanced NSCLC patients (34–43). In this study, an ORR of 83.3%, DCR of 100% and median PFS of 8.3 months were observed in Cohort A (driver gene negative, treat-naïve NSCLC patients tested positive for EGFR protein). These findings demonstrate that addition of SMET12 to immunotherapy plus chemotherapy resulted a potential superior efficacy over the standard combination regimen. However, further clinical trials with increased sample size in cohorts are needed to validate the findings from this study and the clinical benefits from the triple therapy, because of the limited sample size in this study.

Currently, docetaxel alone or combined with anti-angiogenic agents is the standard treatment for advanced NSCLC patients acquiring resistance to immunotherapy (44, 45). Previous studies demonstrated that docetaxel as a second-line treatment achieved an ORR of 7.1% to 24% and median OS of 7 months among NSCLC patients with a prior platinum-based chemotherapy (46). Results from the BTCRC-LUN15–029 study showed that administration of PD-1 or PD-L1 inhibitors followed by pembrolizumab plus next-line chemotherapy (docetaxel or gemcitabine) achieved median PFS of 5.1 months, median OS of 24.5 months and ORR of 23.5% among advanced NSCLC patients with a clinical benefit from previous treatment with a PD-1 or PD-L1 inhibitor (47). Results from the phase III SAPPHIRE study showed that sitravatinib plus nivolumab did not significantly improve OS versus docetaxel, achieving an ORR of 15.6% and median PFS of 4.4 months among non-squamous advanced NSCLC patients who experienced disease progression following immunotherapy plus chemotherapy (48). In this trial, SMET12 plus toripalimab and chemotherapy achieved an ORR of 22.2% and median PFS of 4.2 months among driver gene-negative, advanced NSCLC patients following resistance to first-line immune checkpoint inhibitors-based therapy, and our findings showed that immunotherapy re-challenge regimens (anti-PD-1 agent plus SMET12 and chemotherapy) failed to overcome the acquired resistance to immunotherapy among refractory NSCLC patients. This is consistent with the limitation of the PD-1 retreatment strategy, which may be attributed to the complicated mechanisms underlying resistance to immunotherapy (9, 49). Further studies are required to unravel the exact mechanisms underlying drug resistance, and targeted responses to tackle the resistance are required.

Platinum-based doublet chemotherapy alone or in combination with bevacizumab is the current standard treatment for EGFR-mutated advanced NSCLC with acquired resistance to 3rd-generation EGFR-TKIs (50–52), and immunotherapy is becoming the new direction for next-line treatment of NSCLC patients following resistance to EGFR-TKIs, notably among those with unknow mechanisms of resistance (53, 54). Previous clinical trials have evaluated the application of immunotherapy combination regimens among previously EGFR-TKI treated patients with advanced NSCLC (55–57). Results from both the Checkmate-722 and Keynote-789 study showed that immunotherapy combined with chemotherapy failed to achieve significantly improved PFS over mono-chemotherapy, with median PFS of both 5.6 month (56, 58). Results from the ORIENT-31 study showed that sintilimab plus chemotherapy and bevacizumab significantly improved median PFS over sintilimab plus chemotherapy (7.2 vs. 5.5 months) (59). Results from the HARMONi-A study showed that ivonescimab, a tetravalent bispecific antibody targeting PD-1 and VEGF-A, plus chemotherapy significantly improved median PFS over chemotherapy alone (7.1 vs. 4.8 months) among NSCLC patients with failure in EGFR-TKIs therapy (60). Nevertheless, the satisfactory responses of these two clinicals were both results from immunotherapy combined with anti-angiogenic agents plus chemotherapy. Notably, our findings showed that SMET12 plus toripalimab and chemotherapy achieved an ORR of 41.7%, DCR of 100% and median PFS of 7.2 months, which was comparable to the efficacy reported from the ORIENT-31 study (59) and the HARMONi-A study (60). Our study preliminarily validated the clinical efficacy of this novel triple regimen for treatment of EGFR-mutated, advanced NSCLC patients harboring resistance to targeted therapy. The underlying mechanisms may be that SMET12 specifically binds to EGFR on the surface of cancer cells and CD3 on the surface of lymphocytes to trigger spatial locations of effector T cells near cancer cells and the subsequent generation of immunological synapse, thus activating effector T cells. The PD-1 inhibitors and chemotherapy combinations may exhibit a synergistic effect via triple mechanisms of target modulation, immune activation, and cytotoxicity, thereby presenting a multi-dimensional synergistic antitumor activity. To further validate the efficacy of this triple regimen for treatment of EGFR-mutated NSCLC patients with resistance to targeted therapy, a multi-center, phase 3, randomized, clinical trial seems justified.

Safety is also a major endpoint of this phase 2 clinical trial. Our data showed that 37.5% of the study subjects presented grade 3 or 4 adverse events, and no neurological or grade 5 adverse events were observed. During the treatment period, the most common adverse event was cytokine release syndrome (53.13%), which was associated with SMET12 treatment; 94.12% of cytokine release syndrome was evaluated as grade 1, with no grade 3 identified, and 70.59% occurred within 24 hours of the first treatment with SEMT12. The three most common syndromes included fever (38°C and higher body temperature, 82.4% incidence), chills (11.76%) and hypotension and oxygen deficiency (5.88%). All patients were given supportive therapy, including ibuprofen, promethazine, acetaminophen, glucocorticoid, intravenous fluids, and oxygen inhalation given alone or in combinations, and all recovered within 24 hours post-treatment. The incidence of CRS was 66.67% in cohort A, 36.36% in cohort B and 58.33% in cohort C. Our findings showed the highest incidence of CRS in cohort B, this may be attributed to subjects in cohort B consisting of individuals acquired resistance to chemotherapy and immunotherapy. These patients had suppression of immune functions and poor immune responses; and therefore, the incidence of CRS was low. Conversely, chemotherapy or immunotherapy was not given in other two cohorts, and patients in these two cohorts had good general conditions, which had stronger immune responses than those in cohort B. Therefore, the incidence of CRS was relatively higher in cohorts A and C than in cohort B. In addition, immune-related adverse events included immune-related pneumonia (9.38%), immune-related hepatitis (6.25%), elevated thyroid stimulating hormone (3.13%) and hypothyroidism (3.13%). Taking together, these data demonstrate the tolerable toxicity of this triple regimen.

As an important component of the host immune system, peripheral blood lymphocytes play a vital role in the host anticancer activity (61). Previous studies have shown that peripheral blood lymphocyte counts, activity and subsets are strongly associated with the response to antitumor immunotherapy (62, 63).

In this study, we detected the immune exhaustion status in peripheral blood T cells in three cohorts at baseline, and found the highest degree of immune cell exhaustion in cohort B, followed by in cohort C and A. The variation in immune exhaustion may be closely associated with clinical features of cohorts. Subjects in cohort A were treatment-naïve NSCLC patients negative for driver genes, who had relatively entire immune functions and presented the mildest immune exhaustion status (lowest proportions of Treg cells, PD-1^+^ T cells and LAG-3^+^ T cells). This may be responsible for the superior response to the combination therapy in the cohort A. Participants in cohort B were NSCLC patients with failure in chemotherapy and immunotherapy, and sustained exposure to previous therapy may result in immunosuppression and severe T-cell exhaustion. This regimen has a limited effect on recovery of anti-cancer immune functions. Cohort C consisted of EGFR-mutated patients NSCLC acquiring resistance to targeted therapy. Notably, EGFR-mutated NSCLC is generally considered as an immune-cold tumor, and previous studies have shown that EGFR-mutated NSCLC patients suffer from intrinsic immunologic derangements that are characterized by CD8^+^ T cell exhaustion, elevation of immunosuppressive cytokines and increased counts of Treg cells (53), which is in agreement with the finding from this study that the baseline level of immune exhaustion is higher in cohort C than in cohort A. In addition, detection of baseline immune exhaustion markers revealed that the variation in responses to treatment regimens is associated with the intrinsic difference in baseline immune functions in cohorts. These findings partially unravel the inter-group variation in the incidence of CRS, with highest incidence of CRS in cohort A, followed by in cohorts C and B.

Our findings showed that the peripheral blood lymphocyte counts and counts of activated T cells were significantly among patients with PR than among those with SD, and lower proportions of PD-1^+^ T cells, LAG-3^+^ T cells and Treg cells were detected among patients with PR than among those with SD. Flow cytometry detected higher peripheral blood lymphocyte counts and lower proportions of Treg cells among patients with clinical benefit(CB) than among those with no clinical benefit (NB) at baseline and during the treatment period, and lower proportions of PD-1^+^ T cells and LAG-3^+^ T cells were detected among patients with CB than among those with NB during the treatment period. In addition, the proportions of peripheral blood CD4^+^ Tcm and Tn cells were higher among patients with CB than among those with NB, and the proportions of CD4^+^ Tem and Tte cells were lower among patients with CB than among those with NB. These results indicate potential associations of T lymphocyte counts, subsets and activation with responses to antitumor treatment. Our findings demonstrate that the proportion of CD4^+^ memory T cells may be an important predictor of the therapeutic efficacy. Elevated proportions of CD4^+^ Tcm and Tn cells may facilitate the maintenance of persistent antitumor immune responses, and elevated proportions of Tem and Tte cells may be associated with immune exhaustion, thereby affecting the benefits from the triple regimen.

During the dynamic monitoring of lymphocyte counts, we found that chemotherapy was the main cause responsible for reduced lymphocyte counts. Following three cycles of chemotherapy, the lymphocyte counts appeared a tendency towards a gradual rise among all patients, and patients with PR had their immune functions recovered to exceed baselines. These findings demonstrate that the recovery of immune functions may be associated with therapeutic efficacy, suggesting that the dynamic monitoring of immune functions may serve as a biological marker to assess the response to treatment. In addition, a systematic evaluation of the dynamic effect of chemotherapeutics on immune functions is required during the formulation of the immunotherapy-chemotherapy combination regimen.

## Limitations

This study has some limitations. First, as a single-center, phase II trial, this study has a small sample size, which may result in selective bias, and therefore, the interpretation of the findings from this trial should be prudent. Second, no controls were designed in the trial, which failed to unravel the exact mechanisms underlying the impact of SMET12 on peripheral blood lymphocyte subsets, activity, and subset distributions. Third, OS was unavailable until the data cutoff time because of inadequate follow-up period. Further multi-center, large-scale, randomized, controlled trials are needed to validate the findings from this trial.

## Conclusions

In summary, this phase 2 clinical trial demonstrates for the first time that the combination of the EGFR/CD3 bispecific antibody SMET12, toripalimab, and chemotherapy exhibits a favorable safety profile in advanced NSCLC patients expressing EGFR protein, and demonstrates promising efficacy in EGFR-mutated advanced NSCLC patients with acquired resistance to TKIs. The functional phenotype and differentiation pattern of peripheral blood T lymphocytes exert a critical impact on the therapeutic response to this triple therapy.

## Data Availability

The raw data supporting the conclusions of this article will be made available by the authors, without undue reservation.
