# Supplementary material for: Efficacy and safety of SMET12 in combination with toripalimab and chemotherapy in advanced non-small-cell lung cancer patients tested positive for EGFR protein who are treatment-naïve or harbor acquired resistance to standard therapy: a phase 2, multi-cohort clinical trial
Source: Front Immunol. 2026 Jan 8;16:1706961. doi: 10.3389/fimmu.2025.1706961 (PMC12823912; doi:10.3389/fimmu.2025.1706961)
Supplement: Supplementary file 1 [file DataSheet1.pdf]

Cytokine Assay (pg/mL)

No.	Cohort	Screening ID	Sampling Time	IL-2	IL-4	IL-6	IL-10	TNF-a	IFN-γ	IL-17a
1	B	101008	Baseline	0	0	5.51	0.89	0	0	0
2	B	101003	Baseline	0	0	0	2.14	0	0	0
3	A	101009	Baseline	0	0	10.42	2.62	0	0	0
4	C	101007	Baseline	0	0	0	1.07	0	0	0
5	B	101011	Baseline	0	0	0	0.9	0	0	0
6	B	101010	Baseline	0	0	0.28	5.05	0	0	0
7	B	101013	Baseline	0	0	0	1.12	0	0	0
8	B	101014	Baseline	0	0	0	0	0	0	0
9	B	101015	Baseline	0	0	7.31	1.28	0	0	0
10	C	101016	Baseline	0	0	0.37	0.06	0	0	0
11	C	101017	Baseline	0	0	2.04	1.1	0	0	0
12	B	101019	Baseline	0	0	1.94	1.44	0	0	0
13	B	101020	Baseline	0	0	0	1.23	0	0	0
14	C	101018	Baseline	0	0	0.98	1.66	0	0	0
15	A	101009	C1D17	0	0	46.04	11.99	0	0	0
16	B	101008	C1D17	0	0	11.62	5.08	0	0	0
17	C	101007	C1D17	0	0	16.52	36.07	0	0	0
18	B	101003	C1D17	0	0	3.25	4.9	0	0	0.2
19	B	101011	C1D17	0	0	10.28	7.03	0	0	0.01
20	C	101022	Baseline	0	0	0	1.3	0	0	0
21	B	101013	C1D17	0	0	10.65	24.86	0	0.39	1.32
22	C	101005	C1D17	0	0	4.49	93.68	0	0	0.07
23	B	101004	C1D17	0	0	34.25	1.23	0	0	0.06
24	B	101014	C1D17	0	0	4.8	0.96	0	0	0.36
25	B	101006	C1D17	0	0	2.24	2.01	0	0	0
26	A	101025	Baseline	0	0	1.87	0.96	0	0	0
27	C	101023	Baseline	0	0	2.04	1.05	0	0	0
28	A	101027	Baseline	0	0	50.19	2.19	0	0.21	0.53
29	B	101015	C1D17	0	0	0.73	4.96	0	0	0.09
30	A	101028	Baseline	0	0	7.29	0.88	0.17	0	1.13
31	C	101017	C1D17	0	0	6.31	7.41	0	0	0
32	C	101018	C1D17	0	0	3.98	12.87	0	0	0
33	C	101016	C1D17	0	0	2.04	3.44	0	0	0
34	C	101029	Baseline	0	0	0	0.19	0	0	0
35	A	101030	Baseline	0	0	2.04	3.32	0	0	0
36	C	101022	C1D17	0	0	2.91	1.53	0	0	0
37	A	101002	C3D17	0	0	2.38	0.6	0	0	0
38	A	101031	Baseline	0	0	13.12	0.43	0	0	0

Cytokine Assay（pg/mL）

No.	Cohort	Screening ID	Sampling Time	IL-2	IL-4	IL-6	IL-10	TNF-a	IFN-γ	IL-17a
39	A	101032	Baseline	0.13	0	7.43	1.67	0	0	0
40	A	101001	C3D17	0.31	0	1.69	5.16	0.02	0.14	0
41	C	101023	C1D17	0.09	0	7.03	8.56	0	0.5	0
42	A	101025	C1D17	0.08	0	5.35	5.38	0.02	0	0
43	A	101028	C1D17	0	0	1.95	3.1	0	0	0
44	B	101004	C3D17	0.41	0	18.64	1.91	0	0.3	0
45	C	101029	C1D17	1.27	0	13.9	10.04	0	0.71	0
46	A	101030	C1D17	0	0	5.21	1.25	0	0	0
47	B	101006	C3D17	0.15	0	0.4	10.35	0	0	0
48	C	101005	C3D17	0.19	0	1.3	16.33	0	0	0
49	A	101032	C1D17	0.1	0	58.22	2.91	0	4.07	0
50	C	101007	C3D17	0	0	5.87	7.55	0	0.76	0
51	B	101008	C3D17	0.29	0	4.12	0.9	0	0	0
52	C	101033	Baseline	0.07	0	5.28	1.92	0	0	0
53	A	101009	C3D17	0	0	2.68	3.79	0	0	0
54	B	101014	C3D17	0.06	0	3.82	2.29	0	1.53	0
55	B	101011	C3D17	0.14	0	3.12	3.71	0	0	0
56	C	101018	C3D17	0	0	1.02	4.6	0	0	0
57	B	101013	C3D17	0.17	0	0.14	5.95	0	0.05	0
58	B	101015	C3D17	0.07	0	0.84	1.75	0	0	0
59	B	101003	C3D17	0.35	0	4.34	15.43	0	0	0
60	C	101017	C3D17	0.07	0	27.67	118.52	0	0.23	0
61	C	101033	C1D17	0.33	0.1	7.5	13.65	0.39	0.22	0.83
62	C	101016	C3D17	0	0	0.11	0.74	0.01	0	0
63	C	101023	C3D17	0	0	2.46	1.9	0	0	0
64	C	101022	C3D17	0	0	0	2.09	0	0	0
65	C	101034	Baseline	0	0	2.59	0.27	0	0	0
66	C	101029	C3D17	0.33	0	1.69	1.98	0	1.14	1.64
67	A	101028	C3D17	0.01	0.07	1.92	2.88	0	0	0
68	A	101025	C3D17	0.05	0	4.35	0.13	0	0	0
69	C	101035	Baseline	0	0	0.81	0.6	0	0	3.23
70	A	101030	C3D17	0.09	0	0.43	1.92	0	0	0
71	C	101036	Baseline	0.07	0	0.7	0.38	0	0	0

Cytokine Assay (pg/mL)

No.	Cohort	Screening ID	Sampling Time	IL-2	IL-4	IL-6	IL-10	TNF-a	IFN-γ	IL-17a
72	C	101034	C1D17	0	0	3.81	2.24	0	0	0
73	C	101036	C1D17	0.05	0.12	2.05	7.89	0	0	0
74	C	101035	C1D17	0	0	13.09	63.4	0.53	0	0
75	C	101034	C3D17	0	0	3.86	2.1	0	0	0
76	C	101035	C3D17	0	0	0.43	0.25	0	0	0.06
77	A	101001	6 month	33.14	0	13.66	0.46	0	0	0
78	C	101005	6 month	0	0	0.36	0.33	0.46	0	0
79	C	101007	6 month	0	0	0.72	0.23	0	0	0
80	B	101011	6 month	0.15	0	0.51	0.26	0	0	0
81	B	101013	6 month	0	0	0.98	0.46	0	0	0
82	C	101033	C3D17	0.38	0.19	1.49	1.51	1.16	0	0
83	C	101018	6 month	0	0.11	0.91	0.91	0	0.06	0.28
84	C	101022	6 month	0.02	0	0.67	0.36	0.26	0	0
85	C	101016	6 month	0.03	0	1.07	0.23	0	0	0
86	C	101017	6 month	0	0	5.23	0.33	0	0	0
87	B	101014	6 month	0	0	4.41	0.2	0.02	0	0.42
88	C	101036	C3D17	0	0.02	2.75	0.3	0	0	0
89	A	101028	6 month	0.06	0	1.11	0.12	0	0	0
90	A	101025	6 month	0	0	1.26	0.05	0	0	0
91	C	101023	6 month	0	0.01	4.96	0.16	0	0	0
92	A	101030	6 month	0	0.05	1.07	0.46	0	0	0
93	C	101033	6 month	0.05	0	17.73	0.79	0.27	0	0
94	C	101005	12 month	0.09	0	0.34	0.1	1.04	0	0
95	C	101007	12 month	0	0	1.28	0.06	0	0	0
96	C	101016	12 month	0	0	0.36	0.05	0	0	0
97	B	101011	12 month	0	0	0.31	0.16	0	0	0
98	A	101028	12 month	0	0	0.84	0.02	0	0	0
99	A	101030	12 month	0.11	0	12.9	0.32	0	0	0
100	C	101023	12 month	0	0	7.33	0.06	0	0	0
101	C	101035	12 month	0.15	0	1.72	0.18	0	0	0
